# The biosocial correlates and predictors of emotion dysregulation in autistic adults compared to borderline personality disorder and nonclinical controls

**DOI:** 10.1186/s13229-023-00580-3

**Published:** 2023-12-18

**Authors:** Doha Bemmouna, Amine Lagzouli, Luisa Weiner

**Affiliations:** 1 Department of Psychology, University of Strasbourg, 12 Rue Goethe, 67000 Strasbourg, France; 2 MSME, CNRS UMR 8208, Paris-Est Créteil University, Gustave Eiffel University, 94010 Créteil, France; 3 School of Chemistry, Physics and Mechanical Engineering, Queensland University of Technology, Brisbane, QLD 4001 Australia; 4 Psychiatry Department, University Hospitals of Strasbourg, 1 Place de l’Hôpital, 67000 Strasbourg, France

**Keywords:** Autism spectrum condition, Borderline personality disorder, Emotion dysregulation, Non-suicidal self-injury, Suicidality, Biosocial, Aetiology

## Abstract

**Background:**

Emotion dysregulation (ED) is a core symptom of borderline personality disorder (BPD), whose aetiology has been attributed to biosocial factors. In autism spectrum condition (ASC), although ED is prevalent and is associated with decreased well-being (e.g. self-harm, suicidality), it has been understudied, especially in adults. It is therefore crucial to further understand ED in autistic adults to improve its treatment. Our study investigates ED, its behavioural correlates (e.g. self-harm, suicidality) and biosocial predictors in autistic adults relative to BPD and nonclinical controls (NC).

**Methods:**

A total of 724 participants (ASC = 154; BPD = 111; NC = 459) completed 11 self-reported questionnaires assessing ED, ASC and BPD traits, co-occurring disorders, alexithymia, emotional vulnerability and invalidating experiences (e.g. bullying, autistic camouflaging). The occurrence of ED behavioural correlates (i.e. self-harm, history of suicide attempts, and psychiatric hospitalizations) was collected. In addition, between-groups analyses, linear regressions and machine learning (ML) models were used to identify ED predictors in each group.

**Results:**

ED and its behavioural correlates were higher in ASC compared to NC, but milder than in BPD. While gender did not predict ED scores, autistic women had increased risk factors to ED, including sexual abuse and camouflaging. Interestingly, BPD traits, emotional vulnerability and alexithymia strongly predicted ED scores across the groups. Using ML models, sensory sensitivity and autistic camouflaging were associated with ED in ASC, and ADHD symptoms with ED in BPD.

**Limitations:**

ASC and BPD diagnoses were self-reported, which did not allow us to check their accuracy. Additionally, we did not explore the transactional and the moderating/mediating relationships between the different variables. Moreover, our research is cross-sectional and cannot draw conclusions regarding the direction and causality of relationships between ED and other clinical dimensions.

**Conclusions:**

ED and its behavioural correlates are heightened in BPD compared to ASC and nonclinical controls. In the ASC group, there were no gender differences in ED, despite the heightened exposure of autistic women to ED risk factors. BPD traits, emotional vulnerability, and alexithymia are core to ED regardless of diagnosis. Although less central, sensory sensitivity and autistic camouflaging seem to be specific predictors of ED in autistic adults.

**Supplementary Information:**

The online version contains supplementary material available at 10.1186/s13229-023-00580-3.

## Background

Emotion dysregulation (ED) is defined as patterns of emotional experience or expression that interfere with goal-directed behaviour [1]. ED is recognized as a core etiological and maintenance mechanism of borderline personality disorder (BPD) [2, 3]. Recently, ED has been found to be a key transdiagnostic mechanism of psychopathology involved in the development and maintenance of several psychiatric disorders, such as depression, eating disorders and complex post-traumatic stress disorder (cPTSD) [4–8]. Interestingly, in recent years, ED has also become a central area of research in autism spectrum condition (ASC)^1^ [9, 10]. Indeed, findings support that ED is more prevalent in autistic people compared to the general population [9–12]. Of particular importance, recent findings have reported a high prevalence of self-harm [13–15] and suicidality in ASC [16–18], particularly in autistic adults without intellectual disability presenting with high levels of ED [19–21]. Indeed, similar to findings in BPD and in the general population [14, 22], ED has been associated with self-harm with or without suicidal intent in ASC [19, 21]. Interestingly, akin to BPD [22], self-harm and suicidal behaviours have also been found to be strongly linked in ASC [14, 23]. This suggests that ED is a risk factor for suicidality and self-harm in ASC, and that autistic people may develop capability for suicide through self-harm [14, 23]. Autistic women in particular have been reported to be at greater risk of developing severe ED compared to autistic men [24–26], which suggests that gender-related factors might be involved in ED in ASC [27]. Beyond the fact that ASC and BPD may share ED and ED-related dysregulated behaviours, it is worth noting that ASC and BPD may also co-occur with a pooled prevalence of BPD in ASC of 4% [95% CI 0–9%] and of 3% [95% CI 1–8%] for ASC in BPD [28].

Few studies have focused on interventions targeting ED in autistic adults. Pharmacological treatments have shown limited effectiveness in this context [29]. Therefore, there is a critical need to develop psychological interventions targeting ED in autistic people given the multitude of downstream effects on adaptive functioning and quality of life [30]. Interventions based on cognitive behavioural therapy (CBT) have shown encouraging outcomes [31]. This is particularly the case for dialectical behaviour therapy (DBT) [32, 33], the most studied treatment targeting ED in BPD [22, 34]. Recently, DBT has proved to be feasible and acceptable in autistic adults without intellectual disability, as well as potentially effective to reduce ED in the presence of self-harm and suicidal behaviours [32, 33].

Linehan’s biosocial theory, which underlies DBT, conceptualizes that biological and environmental factors in childhood are involved in the emergence of ED in BPD [34]. According to this theory, ED emerges from an interaction between: (a) emotional vulnerability that stems from biological factors impacting the functioning of brain areas (e.g. prefrontal regions and amygdala) involved in emotional processing, and (b) an invalidating environment that refers to chronic and inadequate responses of the environment to the emotional needs of the child (i.e. neglect, minimization or punishment, including physical and sexual abuse) [22, 34]. Temperamental impulsivity has been subsequently added to the model as an additional risk factor for BPD [22, 35]. According to Linehan, invalidation early in life maintains and may exacerbate the pre-existing biological vulnerability in the child [34]. It also shapes maladaptive coping responses, such as using self-harm with or without suicidal intent when experiencing emotional pain [22, 36]. Most empirical findings support the relevance of Linehan’s model to conceptualize ED in BPD (e.g. [37–41]).

Although DBT has been adapted to and studied in clinical conditions other than BPD (e.g. [42–44]), few studies have focused on the pertinence of Linehan’s biosocial model to conceptualize ED beyond BPD. Nevertheless, there are findings that support the implication of emotional vulnerability [45], including temperamental impulsivity [46], and invalidation (e.g. childhood maltreatment) [47–49] in the development of ED across psychopathology.

In ASC, studies investigating the factors involved in the emergence of ED have mainly focused on ASC-related particularities (e.g. social cognition peculiarities, sensory sensitivity, cognitive inflexibility) [9, 50] and the role played by co-occurring disorders (e.g. anxiety, depression) [11, 20, 51–53]. Thus, to our knowledge, there is a lack of comprehensive models which integrate biological and psychosocial factors potentially involved in the emergence of ED in ASC (e.g. [21, 54–56]).

Based on the existing literature, including Mazefsky and White’s model for ED in autistic youth [50], we recently proposed an application of Linehan’s biosocial model to ED in ASC [57] to provide a comprehensive conceptualization of the biosocial factors (i.e. emotional vulnerability and invalidating environment) involved in the emergence of ED in ASC. More specifically, we considered the interplay between ASC traits and both emotional vulnerability and the experience of invalidation. Indeed, in addition to the biological vulnerability similar to that found in BPD (i.e. hypersensitivity, hyperreactivity and slow return to emotional baseline) [58], theory of mind (ToM) peculiarities, sensory sensitivity, lack of cognitive flexibility, change-related anxiety and repetitive behaviours have been associated with ED in ASC [11, 50]. Alexithymia, prevalent in ASC, has also been reported to be linked to ED in autistic adults [20], especially in autistic women [27]. Moreover, ASC-related difficulties seem to interfere directly with the ability to self-regulate [10, 11, 50], but also contribute to high levels of anxiety and fatigue making emotion regulation costly for autistic people [11, 50, 59]. In terms of invalidating experiences, autistic children are highly exposed to different early stressful and traumatic experiences (e.g. physical and emotional maltreatment from caregivers and school bullying), because of their atypical functioning that cause misunderstanding and rejection from others [60–62]. Autistic girls seem to be particularly vulnerable to the experience of adverse events [63, 64]. Among other environmental factors potentially involved in the emergence of ED, lack of parental scaffolding and modelling (i.e. support provided by the parent to help the child regulate their emotions) have been pinpointed as risk factors for dysregulated behaviours in autistic youth [65, 66]. Additionally, recent studies have reported that autistic camouflaging, i.e. behaviours and/or strategies used to appear “less autistic”, is associated with lifetime suicidality, especially in autistic women [67–69]. Given this, our extension of the biosocial model to ASC includes excessive autistic camouflaging as a form of self-invalidation resulting from internalized invalidation from others [70].

The application of Linehan’s model to ASC requires an empirical test of its validity as well as an assessment of its specificity to ED in ASC, particularly in comparison with BPD. The latter point is crucial, given that ED is highly associated with BPD [71] and that individuals with BPD may exhibit ASC-like traits that may lead to misdiagnosis with ASC [72]. For instance, some studies found that individuals with BPD might also have sensory sensitivity and social cognition peculiarities [73, 74]. Despite the overlap between ASC and BPD, studies comparing ED and its etiological factors in ASC and BPD are lacking.

The aim of the current study is to evaluate the relevance and the specificity of factors involved in Linehan’s model applied to ED in ASC [57]. To do so, autistic adults without intellectual disability (ASC group), adults with BPD (BPD group), and adults without any known diagnoses (nonclinical controls group; NC group) completed a battery of self-report scales measuring the model’s components and indicated the occurrences of the ED behavioural correlates (i.e. hospitalizations, self-harm and suicidal behaviours). We did not assess parental scaffolding as relevant measures are observational in the context of child-parent interaction [65, 75]. Specifically, we were interested in the characteristics of ED and its behavioural correlates in each clinical group. Given that ED is a core feature of BPD and that self-harm and suicidal behaviours are strongly associated with BPD [2, 3, 22], we hypothesized that ED and its behavioural correlates would be higher in the BPD group compared to the ASC group, whereas both clinical groups would have heightened ED scores compared to the NC group (H1). In addition, since self-harm has been associated with ED in BPD [22] and ASC [14, 20], we expected to find an association between self-harm and suicidal behaviours in both groups (H2). We also expected ED to be a predictor of the presence of self-harm and/or suicidal behaviours in both clinical groups (H3). Additionally, in the autistic group only, we expected to observe gender differences, with autistic women presenting with higher ED than autistic men (H4) [27]. Regarding ED predictors, we hypothesized that emotional vulnerability, impulsivity and invalidation—which are direct measures of the biosocial model—would predict ED in both clinical groups, but ASC-related factors would be specific predictors of ED in the ASC group compared to the BPD group (H5). To assess the specific load of ED predictors, machine learning (ML) models were used, and we expected emotional vulnerability and invalidation to emerge among the largest contributors of ED in both clinical groups, while ASC-related factors were expected to rank among the largest ED predictors for the ASC group only (H6).

## Methods

### Participants’ recruitment and study sample

This cross‐sectional study was conducted online from January 2, 2023, to April 26, 2023. Data were collected anonymously through LimeSurvey using a 45-min battery of standardized scales. The call for participants was advertised by different means: emails, leaflets distribution, poster display in mental health institutions, posts on social networks (Facebook, LinkedIn and Discord), targeting various communities: mental health professionals (psychologists and psychiatrists) in institutions or in private practice, researchers in the field of adult ASC and/or BPD, associations in the field of adult ASC or BPD, student communities, and people from the general population more broadly. The call for participants was advertised mainly in France.

The inclusion criteria were: being aged at least 18 years old and being fluent in French. For the ASC and BPD groups, having received a formal diagnosis by a physician was required. The co-occurrence of ASC and BPD was an exclusion criterion from all study analyses. For the no-diagnosis group (NC), participants were required to have no self-reported psychiatric or neurodevelopmental diagnosis.

The 11 participants with ASC + BPD were excluded to prevent any confounding effects, as we were interested in ED specifically associated with ASC and ED in participants with ASC + BPD could be due to co-occurring BPD. In addition, few autistic adults seem to present with a co-occurring BPD (e.g. pooled prevalence of BPD in ASC of 4% [95% CI 0–9%] in the meta-analysis by May et al. [28]); hence, the ED patterns in this subgroup may not be representative of ED in the autistic population.

The sample size has been estimated using G*Power 3.1.9.7 [76] for the three groups comparison on an effect size of *f* = 0.25, a power of 80%, assuming *α* = 0.05. Consequently, a minimum target sample was set at *N* = 125.

### Measures

#### Emotion dysregulation

*Difficulties in emotion regulation scale-16* (DERS-16) [77]. The DERS-16 is a brief form of the 36-item DERS [78]. The DERS-16 is a self-report scale measuring emotion regulation difficulties. It consists of 16 items grouped into five dimensions: (a) lack of emotional clarity (Clarity), (b) difficulty engaging in goal-directed behaviour when distressed (Goals), (c) impulse control difficulties when distressed (Impulse), (d) limited access to strategies for regulation (Strategies), and (e) non-acceptance of emotional responses (Non-acceptance). Items are rated on a 5-point Likert scale (1 = “almost never” to 5 = “almost always”). Higher scores reflect greater emotion regulation difficulties. There is no validated French version of the DERS-16. However, we took the corresponding 16 items from the French-validated 36-item version by Dan-Glauser and Scherer [79]. The scale showed adequate internal consistency (Cronbach's *α* = 0.92) among a college sample [77]. In the present study, the internal consistency for the total DERS-16 was overall excellent for the total scale (Cronbach’s *α* = 0.95) and for the subscales (Cronbach’s *α* between 0.82 and 0.95).

#### ASC and BPD traits importance

*Autism spectrum quotient short version* (AQ-Short) [80]. The AQ-Short is a brief version of the AQ-50 [81]. The AQ-Short is a self-report scale composed of 28 items that assess core autistic traits in adults. The scale comprises four subscales: (a) social skills, (b) routine, (c) attention switching, and (d) imagination. Items are rated on a 4-point Likert scale (1 = “definitely agree” to 4 = “definitely disagree”). Higher scores indicate higher level of autistic traits. There is no validated French version of the AQ-Short. However, we took the 28 corresponding items from the complete French version validated by Lepage et al. [82]. The AQ-Short total score internal consistency has been reported to be good (Cronbach’s *α* between 0.77 and 0.86) [80]. In the present study, the AQ-Short internal consistency was very good (Cronbach’s *α* = 0.87).

*Short form of the borderline symptom list* (BSL-23) [83], French validation by Nicastro et al. [84]. BSL-23 is a short version of the BSL-95 [85]. BSL-23 is a self-report scale constituted of 23 items, assessing the severity of BPD symptoms and behaviours. Each item is answered on a 5-point Likert scale (0 = “not at all” to 4 = 1 “very strong”): 0–1 point refers to no BPD symptoms, 2–23 points to mild, 24–69 points to moderate, and 70–92 points to severe BPD symptoms. The BSL-23 internal consistency is excellent (Cronbach’s *α* = 0.94) [83]. In the present study, the internal consistency of the BSL-23 was excellent (Cronbach’s *α* = 0.97).

#### Co-occurring disorders

*ADHD self-report scale v1.1 screener* (ASRS v1.1 Screener) [86], French validation by Caci et al. [87], is a 6-item self-report measure of attention deficit hyperactivity disorder (ADHD) symptoms in adults. In this study, the ASRS v1.1 was used as a measure of inattention and impulsivity, potentially involved in the biological component of the biosocial model. Items are rated on a 5‐point Likert scale (0 = “never” to 4 = “very often”). Respondents who endorsed at least four out of six items are considered at “elevated” risk for ADHD. The ASRS v1.1 Screener has demonstrated good psychometric properties in studies with adults (Cronbach’s *α* between 0.63 and 0.72) [86, 88]. In the present study, the ASRS v1.1 Screener’s internal consistency was good (Cronbach’s *α* = 0.79).

*Depression, anxiety and stress scales* (DASS-21) [89], French validation by Nahaboo [90], is a shortened version of the DASS-42 [91]. The DASS-21 is a self-report scale that assesses through 21 items the full range of core symptoms of three affective states: depression, anxiety and stress. Items are rated on a 4–point Likert scale (0 = “did not apply to me at all” to 3 = “applied to me very much”). Scores are summed and multiplied by 2 to create separate seven-item subscales for each dimension. The subscales’ internal consistency ranged between excellent and good in the validation study (Depression *α* = 0.91; Anxiety *α* = 0.84; Stress *α* = 0.90) [89]. In the present study, the DASS-21’s internal consistency was excellent (Depression *α* = 0.95) and ranged from very good to excellent for the subscales (Depression *α* = 0.93, Anxiety *α* = 0.86, Stress *α* = 0.93).

#### Emotional vulnerability

*Emotional vulnerability-child scale self-report* (EV-Child) [92] is a self-report scale that retrospectively assesses emotional vulnerability during childhood. The scale includes items addressing Linehan’s [34] concept of slow return to emotional baseline. The scale encompasses 22 items rated on a 6-point Likert scale (1 = “never” to 6 = “always”). Higher scores indicate higher level of emotional vulnerability during childhood. The scale demonstrated excellent internal consistency in the validation study (Cronbach’s *α* = 0.92). In the present study, internal consistency was excellent (Cronbach’s *α* = 0.94).

#### ASC-related factors contributing to ED

*Camouflaging autistic traits questionnaire* (CAT-Q) [93] is a 25-item, self-report questionnaire that assesses social camouflaging behaviour. Items are rated on a 7-point Likert scale (1 = “Strongly disagree” to 7 = “Strongly agree”). The scale contains three subscales: (a) Assimilation, i.e. strategies used to blend in during social situations, (b) Compensation, i.e. strategies to compensate for ASC-related communication and social difficulties, and (c) Masking, i.e. strategies to appear ‘non-autistic’ in social contexts. Higher scores indicated greater camouflaging. There is no French validation of the scale. However, we have used the scale translated and back translated by our team in an ongoing French validation study (Bureau et al. submitted). The CAT-Q has shown excellent internal consistency (Cronbach’s *α* = 0.94) [93]. In the current sample, the internal consistency for the total scale was very good (Cronbach’s *α* = 0.86), good for Compensation subscale (Cronbach’s *α* = 0.76), acceptable for Masking subscale (Cronbach’s *α* = 0.58) and poor for Assimilation subscale (Cronbach’s *α* = 0.45).

*Sensory processing sensitivity questionnaire-sensory sensitivity subscale* (SPSQ SS) [94] is 16-item, self-report scale that measures high sensitivity to various stimuli. The tool encompasses two subscales: (a) Sensory sensitivity (SS) which assesses sensory processing sensitivity) and (b) Other sensitivity (OS) which assesses sensitivity to emotions and various life experiences. Items are rated on an 11-point Likert scale (0 = “compared to others” to 10 = “much more sensitive than the people around me”). Higher scores indicated greater is the sensitivity. In the current study, we only used the SS subscale. The subscale has excellent internal consistency (Cronbach’s *α* = 0.89) [94]. In the current sample the internal consistency of the SS was acceptable (Cronbach’s *α* = 0.67).

*Eight‑item general alexithymia factor score* (GAFS-8) [95] is a self-report unidimensional scale that measures alexithymia using eight items derived from the Toronto Alexithymia Scale (TAS-20) [96]. The selection of items (TAS-20 items: 1, 2, 6, 9, 11, 12, 13 and 14) has been found to be a more robust measure of alexithymia in both autistic and non-autistic samples [95]. Items are rated on a 5-point Likert scale (1 = “Strongly Disagree” to 5 = “Strongly Agree”). Higher scores indicate higher level of alexithymia. As the measure is recent and has not yet been validated in French, we referred to the corresponding items in the French version of the TAS-20 [97]. In the present sample, the internal consistency of the GAFS-8 was excellent (Cronbach's *α* = 0.92).

#### Invalidation and adverse experiences

*Childhood trauma questionnaire-short form* (CTQ-SF) [98], French validation by Paquette et al. [99], is a 28-item, self-report questionnaire designed to assess five types of maltreatment during childhood: (a) physical abuse, (b) sexual abuse, (c) emotional abuse, (d) physical neglect and (e) emotional neglect; with five items per scale (and three additional “minimization” items, which were not used in the present study). Each item is scored on a 5-point Likert (1 = “never true” to 5 = “very often true”). The total score is calculated as the sum of all items, with higher scores reflecting higher rates of childhood traumatic events. The internal reliability of the CTQ-SF is excellent for the total score (Cronbach’s *α* = 0.95), good to excellent for four dimensions (Cronbach’s *α*, respectively, 0.81–0.86, 0.84–0.89, 0.92–0.95 and 0.88–0.91) and acceptable for physical neglect (Cronbach’s *α* ranging from 0.61–0.78) [98]. In the present sample, internal consistency for the CTQ-SF total scale was very good (Cronbach's *α* = 0.92), very good to excellent for 4 of the subscales (Cronbach's *α* between 0.85 and 0.92) and moderate for physical neglect (Cronbach's *α* = 0.68).

*Assessment of bullying experiences* (ABE) [100] is a questionnaire assessing bullying experiences including those unique of autistic and neurodivergent youth (e.g. verbal teasing about social differences) grouped in four subscales: (a) Verbal, (b) Physical, (c) Relational and (d) Cyber bullying. The ABE comprises 22 items that ask parents to rate the frequency with which their child has experienced specific bullying behaviour on a 6-point Likert scale (0 = “has never happened” to 5 = “weekly or more”). In the current study, we used the ABE as a self-report questionnaire and were interested only in the total score. In our sample, the internal consistency of the ABE was excellent (Cronbach’s *α* = 0.94).

#### Socio-demographic and clinical data

The demographic characteristics of participants collected in this study were gender (Woman/man/other), age (years), country of residency, marital status, professional status, educational status and living situation. The clinical data collected were the neurodevelopmental and/or psychiatric diagnoses received, whether the person had current psychotropic medication or not, as well as ED severity indicators: the presence or not of self-harming behaviours in the previous year, the presence or not of suicide ideation in the previous year, lifetime history of at least one suicide attempt and lifetime history of hospitalization in psychiatry services.

The following statement was displayed at the end of the battery of questionnaires: “If these problems are a source of significant distress, it is advisable to seek professional help. You can contact your general practitioner or psychiatrist/psychologist or any other mental health professional. You can also contact your local Medical and Psychological Center (MPC)”. Following this statement, we also shared the national directory of MPCs in France, as well as the emails of the principal investigators, so that the participants could contact them if they had any questions.

### Statistical analysis

#### Descriptive and comparative analyses

Descriptive and comparative statistical analyses were conducted using Jamovi 2.6.23 [101]. Descriptive results were expressed in means (*M*) and standard deviations (*SD*) for continuous variables and in numbers (n) and percentages for ordinal variables.

To investigate whether a significant interaction exists between the groups (ASC/BPD/NC) and each severity indicator of ED, we used the Chi-square test of association [102]. The same test was used to investigate the association between self-harm and suicidal behaviours.

To analyse the predictive value of the DERS-16 total score for self-harm and suicidal behaviours in the ASC and BPD groups, we performed a bimodal logistic regression [103] with two categories (individuals with self-harming and/or suicidal behaviours and those without self-harming and/or suicidal behaviours).

As our data were not normally distributed, we used the Kruskal–Wallis one-way test [104], a nonparametric alternative to the one-way ANOVA, followed by the Steel–Dwass–Chritchlow–Fligner [105] post hoc test, for pairwise comparisons to assess whether there were significant differences between groups on the scales. Epsilon squared (*ε*^2^) was used to calculate the effect size [106]. *ε*^2^ ≤ 0.05 being considered as small effect size, between 0.06 and 0.13 as moderate effect size, and ≥ 0.14 as large effect size [107].

#### Linear regression analyses

To identify ED predictors in each group (ASC/BPD/NC), we conducted linear regression analyses. The following variables were included in the model: ASC, BPD and ADHD traits (assessing impulsivity), emotional vulnerability in childhood, autistic camouflaging, sensory processing particularities, alexithymia, childhood trauma, school bullying and gender for the autistic group (Woman/man). Only women and men were considered due to the small number of participants of other genders. The ordinary least squares (OLS) method was used to estimate the regression coefficients and determine the best-fit line that minimized the sum of squared residuals. We then extracted the model fits statistics, the estimated coefficients for each predictor, and information about the residuals that help interpret the regression analysis.

#### Machine learning models

To study the importance of the ED predictors, we trained a powerful ML model XGBoost [108] using each one of the three groups. We first separated the data into subsets of 80% for training, 10% for validation and 20% for testing to prepare it for training. We then normalized the numerical attributes using standard scaling to ensure that all features contribute equally to the model's learning process.

We also encoded the categorical attributes using one-hot encoding to effectively represent them in a numerical format. To optimize the performance of the XGBoost model, we conducted hyperparameter tuning using a GridSearch [109]. This involved systematically exploring various combinations of hyperparameters and evaluating the model's performance using a suitable evaluation metric to find the optimal ones.

To assess the generalizability of the trained model, we employed cross-validation [110]. Cross-validation involves dividing the dataset into multiple subsets, or folds, and training the model on a subset while evaluating it on the remaining folds. By averaging the performance across all folds, we obtained a more robust estimate of the model's performance. Furthermore, we used SHapley Additive exPlanations (SHAP) [111] values to assess the significance of each feature in the XGBoost model. By calculating the relative contributions of each feature to the expected result, SHAP values offer a consistent measure of feature relevance. This study gives us insights into the underlying relationships and influences within the dataset and helps understand the relative impact of features on the model's predictions.

Statistical significance was set at *p* values ≤ 0.05 and the trend towards significance threshold was set at 0.08.

## Results

### Sample description

**Fig. 1:**
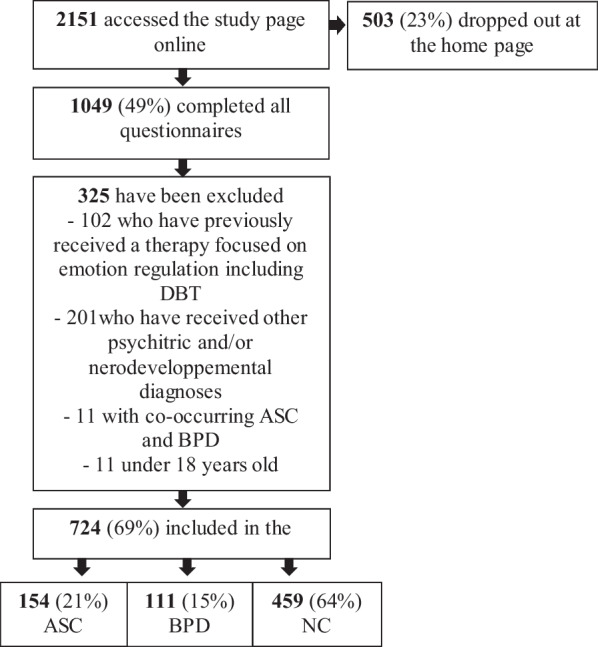
Study flow chart. *Note*: ASC = Autism spectrum condition; BPD = Borderline personality disorder, NC = Nonclinical controls

A total of 2151 individuals began the study and 1049 (49%) provided complete data. 724 (69%) met the inclusion criteria for one of the three groups (Fig. 1). 154 (21%) constituted the ASC group (mean age = 32.62 ± 12.05, range from 18 to 61, 58% women), 111 (15%) constituted the BPD group (mean age = 28.64 ± 8.97, range from 18 to 62, 78% women) and 459 (63%) constituted the NC group (mean age = 25.92 ± 9.43, range from 18 to 65, 75% women) (Table 1). Seventy-nine (11%) participants declared also having a formal diagnosis of ADHD (52 (34%) in the ASC group and 27 (24%) in the BPD group.

**Table 1:** Sample description

	Global	ASC	BPD	NC
*n* (%)	724 (100%)	154 (21%)	111 (15%)	459 (63%)
Mean age (*SD*)	27.77 (10.32)	32.62 (12.05)	28.64 (8.97)	25.92 (9.43)
Age range	18–65	18–61	18–62	18–65
Gender *n* (%)				
Woman	520 (72%)	89 (58%)	87 (78%)	344 (75%)
Man	147 (20%)	46 (30%)	15 (14%)	86 (19%)
Non-binary	57 (8%)	19 (12%)	9 (8%)	29 (6%)
Marital status, *n* (%)				
Single	365 (50%)	83 (54%)	54 (49%)	228 (50%)
Married/in relationship	340 (47%)	62 (40%)	53 (48%)	225 (49%)
Divorced/widow	19 (3%)	9 (6%)	4 (4%)	6 (1%)
Professional status, *n* (%)				
Professionnaly active	184 (25%)	45 (29%)	39 (35%)	100 (22%)
Student	475 (66%)	76 (49%)	50 (45%)	349 (76%)
Unemployed	61 (8%)	33 (21%)	21 (19%)	7 (2%)
Retired	4 (1%)	0 (0%)	1 (1%)	3 (1%)
Educational status, *n* (%)				
High School degree or less	160 (22%)	37 (24%)	37 (33%)	86 (19%)
College graduate	564 (78%)	117 (76%)	74 (67%)	373 (81%)
Living situation, *n* (%)				
Alone	252 (35%)	56 (36%)	45 (41%)	151 (39%)
Alone with children	20 (3%)	6 (4%)	6 (5%)	8 (2%)
With parents	169 (23%)	33 (21%)	23 (21%)	113 (29%)
Flatsharing	89 (12%)	15 (10%)	7 (6%)	67 (17%)
With partner with or without children	194 (27%)	44 (29%)	30 (27%)	49 (13%)
With other psychiatric and/or developmental diagnoses, *n* (%)	205 (28%)	109 (71%)	96 (86%)	0 (0%)
ADHD	79 (11%)	52 (34%)	27 (24%)	0 (0%)
Current psychotropic medication, *n* (%)	172 (24%)	63 (41%)	79 (71%)	30 (7%)

Regarding the co-occurrence of ASC and BPD in our sample, 11 participants reported having been diagnosed with both ASC and BPD, which corresponds to a prevalence of 7% referring to the total number of autistic participants (the ASC group and the 11 ASC + BPD participants we excluded) and of 9% referring to the total number of participants with BPD (the BPD group and the 11 ASC + BPD participants we excluded).

Ninety-nine per cent of participants were from France. The remaining were from other French-speaking countries (two from Belgium, four from Canada and three from Switzerland).

### ED severity indicators

In line with our hypothesis (H1), the BPD group had the highest occurrence of all ED severity indicators (Table 2). Indeed, the prevalence of self-harming behaviours (over the year prior to their participation in the study) in the BPD group was 82% compared to 51% in the ASC group and 35% in the NC group, with these differences being significant (*χ*^2^ *p*_BPD-ASC_ < 0.001, *χ*^2^ *p*_BPD-NC_ < 0.001); the lifetime occurrence of suicide attempts in the BPD group (77%) was significantly higher than in the ASC group (39%) and the NC group (12%) (*χ*^2^ *p*_BPD-ASC_ < 0.001, *χ*^2^ *p*_BPD-NC_ < 0.001); the suicide ideation occurrence was also higher in the BPD group (91%) compared to the ASC group (55%) and the NC group (36%) (*χ*^2^ *p*_BPD-ASC_ < 0.001, *χ*^2^ *p*_BPD-NC_ < 0.001); and the history of hospitalization in psychiatric services was more frequent in the BPD group (64%) than in the ASC group (22%) and the NC group (3%) (*χ*^2^ *p*_BPD-ASC_ < 0.001, *χ*^2^ *p*_BPD-NC_ < 0.001).The ASC group had a higher occurrence of all the ED severity indicators compared to the NC group (*p*_ASC-NC_ < 0.001 for all indicators).

**Table 2:** ED severity indicators occurrence in each group and Chi-square comparison of participant’s distribution between each pair of groups

	ASC *n* = 154	BPD *n* = 111	NC *n* = 459	ASC versus BPD		ASC versus NC		BPD versus NC	
				*χ*^*2*^	*p *value	*χ*^*2*^	*p *value	*χ*^*2*^	*p *value
Past-year self-harm, *n* (%)	79 (51%)	91 (82%)	160 (35%)	26.4	< 0.001^***^	13.1	< 0.001^***^	80.5	< 0.001^***^
Lifetime suicide attempts, *n* (%)	60 (39%)	86 (77%)	54 (12%)	38.7	< 0.001^***^	56.3	< 0.001^***^	208	< 0.001^***^
Self-harm + suicide attempts, *n* (%)	39 (25%)	75 (68%)	33 (7%)	47	< 0.001^***^	36.6	< 0.001^***^	212	< 0.001^***^
Past-year suicide ideation, *n* (%)	85 (55%)	101 (91%)	163 (36%)	39.5	< 0.001^***^	18.5	< 0.001^***^	111	< 0.001^***^
Lifetime psychiatric hospitalization, *n* (%)	34 (22%)	71 (64%)	14 (3%)	47.3	< 0.001^***^	57.8	< 0.001^***^	261	< 0.001^***^

***p < 0.001

In addition, consistent with our hypothesis (H2), a significant association between self-harming behaviours and suicidal behaviours was found in the three groups (*χ*^2^ *p*_BPD_ = 0.008; *χ*^2^ *p*_ASC_ = 0.007; *χ*^2^ *p*_NC_ < 0.001) (Table 3).

**Table 3:** Chi-square test of association between self-harm and suicidal behaviours in the sample and in each group

	Global *n* = 724		ASC *n* = 154		BPD *n* = 111		NC *n* = 459	
	Suicide attempts	No suicide attempts	Suicide attempts	No suicide attempts	Suicide attempts	No suicide attempts	Suicide attempts	No suicide attempts
Self-harm	147 (20%)	183 (25%)	39 (25%)	40 (26%)	75 (68%)	16 (14%)	33 (7%)	127 (28%)
No self-harm	53 (7%)	341 (47%)	21 (14%)	54 (35%)	11 (10%)	9 (8%)	21 (5%)	278 (61%)
*χ*^*2*^	86.8		7.39		7.06		18.6	
*p *value	< 0.001^***^		0.007^**^		0.008^**^		< 0.001^***^	

^**^p < 0.01, ***p < 0.001

### Link between ED and self-harm and/or suicidal behaviours

In line with our hypothesis (H3), the bimodal logistic regression analysis showed that the DERS-16 mean score significantly predicted the presence of self-harm and/or suicidal behaviours in both the ASC group (*z* = 3.700, *p* < 0.011) and the BPD group (*z* = 2.208, *p* = 0.027).

The DERS-16 prediction accuracy rates were 71% for the ASC group and 92% in the BPD group. The prediction accuracy rate of the DERS-16 is especially high for the presence of self-harm and or suicidal behaviours (90% for ASC and 100% for BPD) (Table 4).

**Table 4:** Association between DERS-16 total and self-harm and suicidal behaviours using bimodal logistic regression

Predictor	ASC *n* = 154		BPD *n* = 111	
	*z*	*p *value	*z*	*p *value
DERS	3.7	< 0.001^***^	2.208	0.027^*^
Prediction accuracy rate	71%		92%	
With SH and/or SA	90%		100%	
Without SH and/or SA	37%		0%	

*p < 0.05, ^***^p < 0.001

*SH* self-harm, *SA* suicide attempts

### Questionnaire scores’ comparisons between groups

#### Emotion dysregulation

As hypothesized (H1), the BPD group had significantly higher scores on the DERS-16 total scale and on its subscales than the ASC group, with large effect sizes, except for Clarity and Goals subscales (*W* = 11.75, *p* < 0.001 for the total; *W* = 5.56, *p* = 0.005 for Clarity; *W* = 12.84, *p* < 0.001 for Impulse; *W* = 9.72, *p* < 0.001 for Non-acceptance; *W* = 5.23, *p* = 0.011 for Goals; *W* = 11.00, *p* < 0.001 for Strategies) and the NC group (*W* = 17.03, *p* < 0.001 for the total; *W* = 13.29, *p* < 0.001 for Clarity; *W* = 17.25, *p* < 0.001 for Impulse; *W* = 13.69, *p* < 0.001 for Non-acceptance; *W* = 11.70, *p* < 0.001 for Goals; *W* = 16.75, *p* < 0.001 for Strategies). The DERS-16 total score was significantly higher in the ASC group compared to the NC group (*W* = 7.35, *p* < 0.001). This was also the case for the DERS-16 subscales (*W* = 9.10, *p* < 0.001 for Clarity; *W* = 5.70, *p* < 0.001 for Impulse; *W* = 3.48, *p* = 0.037 for Non-acceptance; *W* = 7.10, *p* < 0.001 for Goals; *W* = 6.86, *p* = 0.002 for Strategies) (Table 5). See additional material for the correlation analyses between the scales for each group [Additional file 1].

**Table 5:** Scales mean scores pairwise comparisons between the groups using the Kruskal–Wallis one-way test

	ASC *n* = 154		BPD *n* = 111		NC *n* = 459		ASC versus BPD			ASC versus NC			BPD versus NC		
	*M*	SD	*M*	SD	*M*	SD	*W*	*p *value	*ε*^2^	*W*	*p *value	*ε*^2^	*W*	*p *value	*ε*^2^
DERS-16	49.86	15.05	65.87	11.7	42.15	16.45	11.75	< 0.001^***^	0.2617^a^	7.35	< 0.001^***^	0.0442	17.03	< 0.001^***^	0.2549^a^
Clarity	6.31	2.24	7.41	2.01	4.94	2.31	5.56	0.005^**^	0.0586	9.10	< 0.001^***^	0.0676	13.29	< 0.001^***^	0.1551^a^
Impulse	7.24	3.51	11.84	3.23	6.17	3.58	12.84	< 0.001^***^	0.3120^a^	5.70	< 0.001^***^	0.0265	17.25	< 0.001^***^	0.2616^a^
Non-acceptance	8.36	3.86	11.76	3.33	7.51	3.82	9.72	< 0.001^***^	0.1789^a^	3.48	0.037^*^	0.0099	13.69	< 0.001^***^	0.1647^a^
Goals	11.82	3.08	13.21	2.16	10.15	3.64	5.23	< 0.001^***^	0.0519	7.10	< 0.001^***^	0.0412	11.70	< 0.001^***^	0.1203
Strategies	16.14	5.91	21.66	3.84	13.38	6.05	11.00	< 0.001^***^	0.2290^a^	6.86	0.002^**^	0.0384	16.75	< 0.001^***^	0.2466^a^
AQ-Short	21.1	3.6	15.41	4.84	12.79	5.66	− 12.68	< 0.001^***^	0.3044^a^	20.26	< 0.001^***^	0.3355^a^	6.20	< 0.001^***^	0.0338
BSL-23	30.91	21.1	60.55	19.09	24.21	21.51	13.48	< 0.001^***^	0.3443^a^	5.48	< 0.001^***^	0.0279	17.72	< 0.001^***^	0.2761^a^
ASRS v1.1 Screener	15.1	5.59	15.95	4.69	12.66	4.96	1.43	0.568	0.0039	7.14	< 0.001^***^	0.0416	8.70	< 0.001^***^	0.0666
DASS-21	56.16	28.56	82.77	23.54	43.16	29.04	10.36	< 0.001^***^	0.2034^a^	6.87	< 0.001^***^	0.0386	15.99	< 0.001^***^	0.2246^a^
Stress	21.99	10.13	30.83	7.63	17.38	10.63	9.96	< 0.001^***^	0.1878^a^	6.64	< 0.001^***^	0.0361	15.61	< 0.001^***^	0.2140^a^
Anxiety	14.73	10.18	23.62	9.99	11.58	9.93	9.45	< 0.001^***^	0.1690^a^	5.21	< 0.001^***^	0.0222	13.99	< 0.001^***^	0.1719^a^
Depression	19.44	12.8	28.32	10.13	14.2	11.69	7.86	< 0.001^***^	0.1169	6.37	< 0.001^***^	0.0332	14.41	< 0.001^***^	0.1824^a^
EV-Child self-report	92.28	20.09	101.42	19.45	74.62	24.1	5.41	< 0.001^***^	0.0555	11.29	< 0.001^***^	0.1042	14.11	< 0.001^***^	0.1749^a^
CAT-Q	100.75	11.86	94.59	13.99	87.87	14.59	− 5.35	< 0.001^***^	0.0542	13.44	< 0.001^***^	0.1476^a^	5.86	< 0.001^***^	0.0302
Compensation	44.08	7.72	39.61	9.67	34.24	10.54	− 5.49	< 0.001^***^	0.0571	14.41	< 0.001^***^	0.1696^a^	6.51	< 0.001^***^	0.0373
Masking	23.09	3.75	21.27	4.46	20.79	3.84	− 5.25	< 0.001^***^	0.0522	9.15	< 0.001^***^	0.0684	1.51	0.536	0.0020
Assimilation	33.58	4.55	33.7	4.34	32.84	4.47	− 2.48	0.983	0.0001	2.91	0.098	0.0069	2.51	0.177	0.0056
SPSQ SS subscale	56.36	10.13	54.65	9.71	49.24	9.17	− 2.38	0.211	0.0108	11.43	< 0.001^***^	0.1051	7.31	< 0.001^***^	0.0470
GAFS-8	29.38	7.99	29.5	7.29	23.24	9.21	− 0.45	0.946	0.0004	10.18	< 0.001^***^	0.0846	9.05	< 0.001^***^	0.0720
CTQ-SF	48.49	16.01	57.77	17.88	40.12	12.87	5.99	< 0.001^***^	0.0679	8.41	< 0.001^***^	0.0577	13.78	< 0.001^***^	0.1669^a^
Emotional abuse	12.57	5.46	15.35	5.5	9.59	4.75	5.59	< 0.001^***^	0.0593	8.93	< 0.001^***^	0.0651	13.49	< 0.001^***^	0.1600^a^
Physical abuse	6.72	3.34	8.35	5.08	6.04	2.54	4.00	0.013^*^	0.0303	3.41	0.042^*^	0.0095	7.91	< 0.001^***^	0.0550
Sexual abuse	7.37	4.35	9.61	6.23	6.29	3.5	3.78	0.021^*^	0.0270	6.21	< 0.001^***^	0.0315	9.73	< 0.001^***^	0.0833
Emotional neglect	13.73	4.99	15.27	4.79	11.27	4.69	3.14	0.068	0.0187	7.58	< 0.001^***^	0.0469	10.40	< 0.001^***^	0.0951
Physical neglect	8.09	3.21	9.19	3.62	6.94	2.49	3.73	0.023^*^	0.0264	5.94	< 0.001^***^	0.0288	9.57	< 0.001^***^	0.0804
ABE	43.75	22.4	43.87	25.05	24.81	18.1	− 0.21	0.988	0.0000	12.93	< 0.001^***^	0.1365	10.47	< 0.001^***^	0.0964

*p < 0.05, ^**^p < 0.01, ^***^p < 0.001

^a^Large effect size

#### ASC and BPD traits

As expected, on the AQ-Short, the score of the ASC group was significantly higher than the BPD group, with large effect sizes (*W* = − 12.68, *p* < 0.001) and the NC group (*W* = 20.26, *p* < 0.001), while the BPD group scored higher than the NC group (*W* = 6.20, *p* < 0.001). On the BSL-23, the BPD group score was significantly higher than the ASC group (*W* = 13.48, *p* < 0.001) and the N.C group (*W* = 17.72, *p* < 0.001) with large effect sizes, while the ASC group scored significantly higher than the NC group (*W* = 5.48, *p* < 0.001).

#### Co-occurring disorders

On the ASRS v1.1, there was no significant difference between the ASC and BPD groups (*W* = 1.43, *p* = 0.568), both scoring significantly higher than the NC group (*W*_ASC-NC_ = 7.14, *p* < 0.001; *W*_BPD-NC_ = 8.70, *p* < 0.001).

On the DASS-21, the score of the BPD group was significantly higher than that of the ASC group (*W* = 10.36, *p* < 0.001) and the NC group (*W* = 15.99, *p* < 0.001) with large effect sizes except. Specifically, the BPD group scored significantly higher on the Stress and Depression subscales (*W* = 9.96, *p* < 0.001 and *W* = 9.45, *p* < 0.001, respectively) compared to the ASC group, and significantly higher on all subscales compared to the NC group. The ASC group scored significantly higher than the NC group (*W* = 6.87, *p* < 0.001).

#### Emotional vulnerability

On the EV-Child, the score of the BPD group was significantly higher than the ASC group (*W* = 5.41, *p* < 0.001) and the NC group (*W* = 14.11, *p* < 0.001), respectively, with small and large effect sizes. The ASC group scored significantly higher than the NC group (*W* = 11.29, *p* < 0.001), with a moderate effect size.

#### ASC-related factors contributing to ED

On the CAT-Q (camouflaging), the ASC group had significantly higher scores than the BPD group (*W* = − 5.35, *p* < 0.001) and the NC group (*W* = 13.44, *p* < 0.001), while the BPD group had a significantly higher score than the NC group (*W* = 5.86, *p* < 0.001). This was also the case for the Compensation subscale (*W*_ASC-BPD_ = − 5.94, *p* < 0.001; *W*_ASC-NC_ = 14.41, *p* < 0.001; *W*_BPD-NC_ = 6.51, *p* < 0.001). On the Masking subscale, the ASC group had significantly higher scores than the BPD group (*W* = − 5.25, *p* < 0.001) and the NC group (*W* = 9.15, *p* < 0.001), but no significant difference was found between the BPD and NC groups (*W* = 1.51, *p* = 0.536). There was no significant difference between the three groups on the Assimilation subscale.

On the SPSQ SS (sensory sensitivity) subscale score, there was no significant difference between the ASC and BPD groups (*W* = − 1.69, *p* = 0.456), both scoring significantly higher than the NC group (*W*_ASC-NC_ = − 6.87, *p* < 0.001; *W*_BPD-NC_ = 4.25, *p* = 0.007).

Similarly, on the GAFS-8 (alexithymia), there was no significant difference between the ASC and BPD groups (*W* = − 2.38, *p* = 0.211), both scoring significantly higher than the NC group (*W*_ASC-NC_ = 11.34, *p* < 0.001; *W*_BPD-NC_ = 7.31, *p* < 0.001).

#### Invalidation and adverse experiences

On the CTQ-SF, the score was significantly higher in the BPD group compared to the ASC group (*W* = 5.99, *p* < 0.001) and the NC group (*W* = 13.78, *p* < 0.001). This was also the case for all the CTQ-SF subscales except for Emotional neglect, although a trend towards significance was observed (*W* = 3.14, *p* = 0.068). The ASC group scored significantly higher than the NC group on the CTQ-SF total (*W* = 8.41, *p* < 0.001) and subscales.

On the ABE, there was no significant difference between the ASC and BPD groups (*W* = − 0.21, *p* = 0.988), both scoring significantly higher than the NC group (*W*_ASC-NC_ = 12.93, *p* < 0.001; *W*_BPD-NC_ = 10.47, *p* < 0.001).

### Gender differences

When considering gender differences (Table 6), we did not include the non-binary (i.e. “other”) subgroup because of the small number of participants (*n* = 19).

**Table 6:** Scales mean scores pairwise comparisons between ASC women, ASC men and BPD using the Kruskal–Wallis one-way test

	ASC women *n* = 89		ASC men *n* = 46		BPD *n* = 111		ASC women versus ASD Men			ASC women versus BPD			ASC Men versus BPD		
	*M*	SD	*M*	SD	*M*	SD	*W*	*p* value	*ε*^*2*^	*W*	*p* value	*ε*^*2*^	*W*	*p *value	***ε***^***2***^
DERS-16	50.98	14.5	46.26	16.39	65.87	11.7	− 2.26	0.248	0.0189	9.93	< 0.001^***^	0.2477^a^	− 9.24	< 0.001^***^	0.2735^a^
Clarity	6.43	2.1	6.04	2.37	7.41	2.01	− 1.41	0.581	0.0074	4.63	0.003^**^	0.0539	− 4.66	0.003^**^	0.0695
Impulse	7.4	3.37	7.09	3.88	11.84	3.23	− 1.26	0.646	0.0059	11.19	< 0.001^***^	0.3149^a^	− 9.05	< 0.001^***^	0.2623^a^
Non-acceptance	8.67	3.67	7.26	3.9	11.76	3.33	− 3.14	0.068^a^	0.0368	8.14	< 0.001^***^	0.1663^a^	− 8.52	< 0.001^***^	0.2328^a^
Goals	12.07	2.91	10.93	3.5	13.21	2.16	− 2.68	0.14	0.0268	3.84	0.018*	0.0370	− 5.91	< 0.001^***^	0.1120
Strategies	16.4	5.85	14.93	6.08	21.66	3.84	− 1.76	0.428	0.0115	9.25	< 0.001^***^	0.2151^a^	− 9.07	< 0.001^***^	0.2638^a^
AQ-Short	21.17	3.56	21.00	3.79	15.41	4.84	− 0.51	0.931	0.0005	− 11.28	< 0.001^***^	0.3165^a^	8.73	< 0.001^***^	0.2447^a^
BSL-23	31.07	19.68	27.3	20.68	60.55	19.09	− 1.87	0.382	0.0131	12.05	< 0.001^***^	0.3647^a^	− 10.29	< 0.001^***^	0.3391^a^
ASRS v1.1 Screener	15.17	5.76	14.52	4.9	15.95	4.69	− 1.36	0.600	0.0069	0.94	0.780	0.0022	− 2.53	0.173	0.0205
DASS-21	59.82	27.51	46.3	27.55	82.77	23.54	− 3.90	0.016^*^	0.0567	8.32	< 0.001^***^	0.1740^a^	− 9.26	< 0.001^***^	0.2748^a^
Stress	24.11	9.65	17.61	9.87	30.83	7.63	− 5.22	< 0.001^***^	0.1018	7.01	< 0.001^***^	0.1234	− 9.88	< 0.001^***^	0.3128^a^
Anxiety	16.38	10.11	10.96	8.14	23.62	9.99	− 4.42	0.005^**^	0.0730	7.02	< 0.001^***^	0.1238	− 9.34	< 0.001^***^	0.2796^a^
Depression	19.33	12.11	17.74	13.88	28.32	10.13	− 1.31	0.625	0.0064	7.36	< 0.001^***^	0.1360	− 6.08	0.005^**^	0.1184
EV-Child self-report	95.58	19.22	86.93	22.58	101.42	19.45	− 3.27	0.054^a^	0.0399	3.29	0.053^a^	0.0271	− 5.31	< 0.001^***^	0.0904
CAT-Q	102.55	11.98	96.65	11.59	94.59	13.99	− 4.01	0.013^*^	0.0600	− 5.83	< 0.001^***^	0.0855	1.63	0.482	0.0085
Compensation	45.21	7.61	41.48	7.78	39.61	9.67	− 3.75	0.022^*^	0.0524	− 5.94	< 0.001^***^	0.0886	1.80	0.409	0.0104
Masking	23.13	3.91	23.04	3.61	21.27	4.46	− 0.48	0.939	0.0009	− 4.80	0.002^**^	0.0579	3.47	0.038^*^	0.0385
Assimilation	34.2	4.42	32.13	4.75	33.7	4.34	− 3.97	0.014^*^	0.0589	− 1.49	0.544	0.0056	− 2.18	0.273	0.0152
SPSQ SS subscale	58.01	9.47	53.13	11.34	54.65	9.71	− 4.31	0.006^**^	0.0695	− 3.91	0.016^*^	0.0384	− 1.22	0.663	0.0048
GAFS-8	29.61	7.58	28.63	8.68	29.5	7.29	− 0.70	0.873	0.0019	− 0.43	0.950	0.0005	− 0.23	0.9086	0.0002
CTQ-SF	49.62	16.2	45.39	14.08	57.77	17.88	− 1.99	0.337	0.0148	4.54	0.004^**^	0.0518	− 5.81	< 0.001^***^	0.1082
Emotional abuse	13.13	5.37	11.15	5.18	15.35	5.5	− 3.03	0.082	0.0342	3.90	0.016^*^	0.0382	− 6.04	< 0.001^***^	0.1168
Physical abuse	6.69	3.73	6.61	2.39	8.35	5.08	1.87	0.381	0.0131	4.45	0.005^**^	0.0499	− 2.04	0.32	0.0133
Sexual abuse	8.11	4.9	5.78	2.5	9.61	6.23	− 5.00	0.001^**^	0.0933	1.46	0.0557^a^	0.0053	− 5.63	< 0.001^***^	0.1016
Emotional neglect	13.7	4.84	13.85	5.06	15.27	4.79	0.37	0.964	0.0005	3.01	0.0850	0.0227	− 1.87	0.384	0.0112
Physical neglect	7.99	2.81	8	3.39	9.19	3.62	− 0.64	0.895	0.0015	3.20	0.062^a^	0.0257	− 3.03	0.082	0.0294
ABE	44.09	22.12	40.5	22.14	43.87	25.05	− 1.36	0.603	0.0069	− 0.41	0.955	0.0004	− 1.001	0.759	0.0032

*p < 0.05, ^**^p < 0.01, ^***^p < 0.001

^a^Trend towards significance

Contrary to our hypothesis (H4), autistic women did not present with higher ED compared to autistic men (*W* = − 2.26, *p* = 0.248).

However, autistic women had a significantly higher DASS-21 score than the autistic men (*W* = − 3.90, *p* = 0.016) and the BPD group (*W* = 8.32, *p* < 0.001), especially regarding the Stress (*W*_ASC-BPD_ = − 5.22, *p* < 0.001 and *W*_ASC-NC_ = 7.01, *p* < 0.001) and Anxiety subscales (*W*_ASC-BPD_ = − 4.42, *p* = 0.005 and *W*_ASC-NC_ = 7.02, *p* < 0.001).

On the EV-Child (emotional vulnerability), the score tended to be higher in autistic women compared to men (*W* = − 3.27, *p* = 0.054). No significant difference was found relative to the BPD group, but there was a trend for a higher score in the BPD group compared to autistic women (*W* = 3.29, *p* = 0.053).

On the CAT-Q (measuring total camouflaging and Compensation subscale), autistic women scored significantly higher than autistic men (*W* = − 4.01, *p* = 0.013) and the BPD group (*W* = − 5.83, *p* < 0.001), while no differences were found between ASC men and the BPD group (*W* = 1.63, *p* = 0.482). On the Assimilation subscale, autistic women scored significantly higher than autistic men (*W* = − 3.97, *p* = 0.014) but similar to the BPD group (*W* = − 1.49, *p* = 0.544).

Autistic women had a significantly higher SPSQ SS (sensory processing) scores than ASC men (*W* = − 4.31, *p* = 0.006) and the BPD group (*W* = − 3.91, *p* = 0.016). There was no significant difference between autistic men and the BPD group on this scale (*W* = − 1.22, *p* = 0.663).

On the CTQ-SF (childhood maltreatment), the sexual abuse subscale score was significantly higher in autistic women than autistic men (*W* = − 5.00, *p* = 0.001) and comparable to the BPD group, although they tended to be lower than the latter group (*W* = 1.46, *p* = 0.056).

### Predictors of ED

#### Linear regression models

Partially supportive of our hypothesis (H5), linear regression analyses showed three significant predictors of ED in the ASC group, with the regression model accounting for 54.6% of the variance (Table 7): BPD traits measured by the BSL-23 (*t* = 7.348, *p* < 0.001), emotional vulnerability measured by the EV-Child (*t* = 3.682, *p* < 0.001) and alexithymia measured by the GAFS-8 (*t* = 3.805, *p* < 0.001). A trend towards significance was found on the SPSQ SS subscale measuring sensory sensitivity (*t* = 1.916, *p* = 0.057).

**Table 7:** Linear regression models for ED predictors

Predictors	ASC *n* = 154		BPD *n* = 111		NC *n* = 459	
	*t*	*p *value	*t*	*p *value	*t*	*p * value
Woman	− 0.409	0.683	− 0.737	0.463	− 0.776	0.438
Man	− 0.583	0.561	− 0.895	0.373	− 0.736	0.462
AQ-short	1.632	0.105	− 0.432	0.666	0.93	0.353
BSL-23	7.348	< 0.001^***^	3.9	< 0.001^***^	13.383	< 0.001^***^
ASRS v1.1 screener	1.501	0.136	1.851	0.067^a^	3.054	0.002^**^
EV-Child self-report	3.682	< 0.001^***^	2.386	0.019^*^	6.091	< 0.001^***^
CAT-Q	0.796	0.428	− 0.469	0.640	− 2.297	0.022^*^
SPSQ SS subscale	1.916	0.057^a^	0.58	0.563	2.603	0.010^*^
GAFS-8	3.805	< 0.001^***^	2.256	0.026^*^	8.017	< 0.001^***^
CTQ-SF	− 1.631	0.105	− 0.205	0.838	− 1.051	0.294
ABE	− 1.002	0.318	0.963	0.338	− 0.027	0.979
*R*^2^	0.579		0.457		0.676	
Adjusted *R*^2^	0.546		0.396		0.668	

*p < 0.05, ^**^p < 0.01, ^***^p < 0.001

^a^Trend towards significance

In the BPD group, the same three variables were found to be strong predictors of ED, with the regression model accounting for 39.6% of the variance: BPD traits (*t* = 3.900, *p* < 0.001), emotional vulnerability (*t* = 2.386, *p* = 0.019) and alexithymia (*t* = 2.256, *p* = 0.026). A trend towards significance was found on the ASRS v1.1 Screener measuring ADHD symptoms (*t* = 1.851, *p* = 0.067).

In the NC group, six significant predictors of ED were found, with the regression model accounting for 66.8% of the variance: BPD traits (*t* = 13.383, *p* < 0.001), ADHD symptoms (*t* = 3.054, *p* = 0.002), emotional vulnerability (*t* = 6.091, *p* < 0.001), autistic camouflaging measured by the CAT-Q (*t* = − 2.297, *p* = 0.022), sensory hypersensitivity (*t* = 2.603, *p* = 0.010) and alexithymia (*t* = 8.017, *p* < 0.001).

#### ED Predictors rankings

**Fig. 2:**
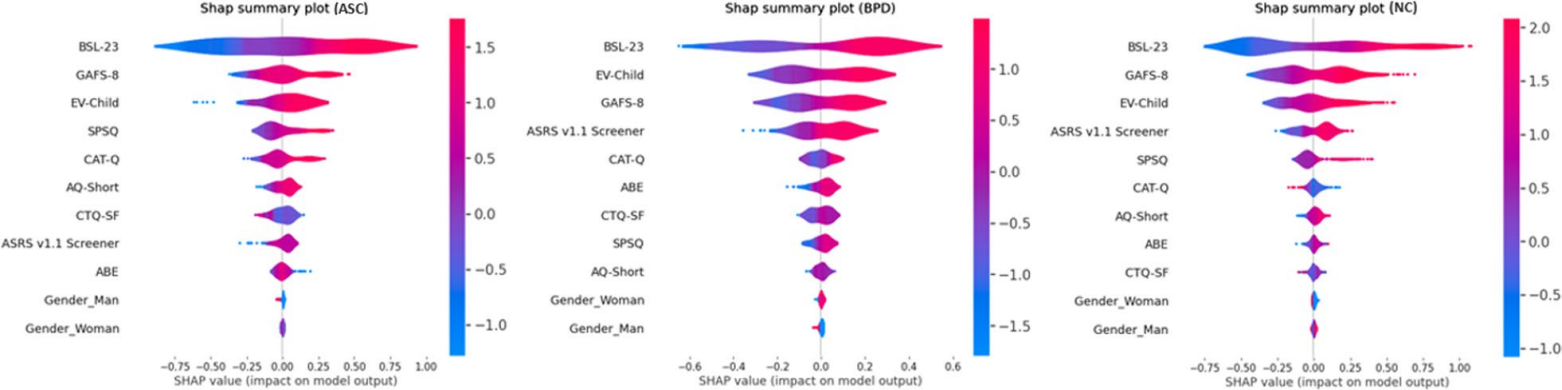
Shapley values plots illustrate how explanatory variables contribute to ED in each group (ASC/BPD/NC). The feature list down the *y*-axis is in order of contribution to the model (most to least). On the *x*-axis, the SHAP values for each observation are presented—negative SHAP values are interpreted as reduced ED, while positive SHAP values are interpreted as increased ED. Each dot represents an individual respondent; hence, the number of dots against each feature reflects the sample size of the training set. The dot’s position along the *x*-axis is the feature’s impact on the model’s prediction for that respondent. The colour indicates whether the value of the characteristic considered is high or low in relation to the range of values (red refers to high values and blue to low values). When multiple dots arrive at the same coordinate in the plot, they pile up to show the density of effect sizes. The graph has a median line and the farther the point is from the median line, the stronger is the influence on the output, with the points on the right correlating positively with ED and the points on the left negatively)

Figure 2 presents the SHAP summary plots that display the importance of the 11 variables selected, the magnitude of their impact (i.e. the effect size) in each group and the direction of a specific feature’s association with ED. For both groups, our hypothesis (H6) was partially supported.

For the ASC group, BPD traits (BSL-23), alexithymia (GAFS-8), emotional vulnerability (EV-Child), sensory sensitivity (SPSQ SS) and autistic camouflaging (CAT-Q), respectively, carried most of the general model’s predictive power with an absolute mean SHAP value of 0.37, 0.14, 0.13, 0.11 and 0.09, respectively.

For the BPD group, BPD traits, emotional vulnerability, alexithymia and ADHD traits (ASRS v1.1 Screener), respectively, carried most of the general model’s predictive power with an absolute mean SHAP value of 0.28, 0.15, 0.13 and 0.10, respectively.

For the NC group, BPD traits, alexithymia, emotional vulnerability, ADHD traits and sensory sensitivity, respectively, carried most of the general model’s predictive power with an absolute mean SHAP value of 0.40, 0.21, 0.15, 0.10 and 0.08, respectively.

For all groups, gender (Woman/Man) ranked at the bottom of the model, indicating a very low predictive value on ED scores.

Furthermore, the SHAP value range for the BSL-23 was wider in the ASC group compared to the BPD group, which means that the BPD traits predicted more strongly ED in the ASC group than in the BPD group.

## Discussion

Our study is the first to provide an investigation of ED in autistic adults compared to BPD and nonclinical controls. Our results suggest that ED scores and its behavioural correlates (i.e. suicidality, self-harming behaviour and hospitalizations) are increased in ASC compared to ND. Nevertheless, people with BPD had the highest scores of ED and the greatest occurrences of its behavioural correlates in our sample. Interestingly, and contrary to our hypotheses, the same three dimensions, i.e. BPD traits, emotional vulnerability, and alexithymia, significantly predicted ED scores in both the ASC and BPD groups. Consistently, these three dimensions ranked as the greatest ED predictors in the ML models for both the ASC and BPD groups, whereas sensory sensitivity and autistic camouflaging were associated with ED in ASC, and ADHD symptoms with ED in BPD.

First of all, in line with our hypothesis (H1), we found that ED was higher in the BPD group than the ASC group, with both clinical groups scoring higher than the nonclinical controls. This result is not surprising given that ED is a core feature of BPD [2, 3], and that ED is typically considered as a co-occurring difficulty in autistic people [50]. Yet, unlike our results, recent findings by Weiner et al. [27] suggested that ED was heightened in autistic adults, particularly autistic women, compared to women with BPD. However, their sample of autistic people was recruited from a DBT waiting list; hence, their results could not be generalized to autistic adults in other contexts. This is not the case for our results, as both the BPD and the ASC groups were recruited from the general population.

Relatedly, consistent with our hypothesis (H1), the prevalence of suicidal behaviour, use of psychiatry services and self-harm was higher in the BPD group compared to the ASC group, while these behaviours were more frequent in both clinical groups compared to the NC group. Interestingly, the rates of suicidal and self-harming behaviours found in the BPD and ASC groups are consistent with previous findings. Indeed, in their review, Oumaya et al. [22] reported that self-harm rates ranged between 50 and 80% and suicidality rates ranged between 40 and 85% in BPD, which match the 82% prevalence of self-harm and 77% of suicidal behaviours in our BPD group. In our ASC group, the rate of 41% of self-harm matches the 50% reported by Maddox et al. [19], and the rate of 39% of suicide attempts is congruent with the 35% rate found by Cassidy et al. [112]. These results add to previous research showing that the rates of self-harm and suicidal behaviours in autistic adults are higher than that of non-autistic people [13, 14]. Nevertheless, our study is the first to show that the prevalence of these behavioural ED correlates is decreased in ASC compared to BPD. These results are congruent with the ED levels found in our groups. Indeed, while ED scores are higher in the ASC group compared to the NC group, the BPD group displayed the highest ED scores in our sample. Moreover, consistent with our hypotheses (H2 and H3**)**, self-harm and suicidal behaviours were strongly associated in each group and ED strongly predicted these behaviours in both clinical groups. This is in line with numerous studies, suggesting that there is a strong link between self-harm and suicidality [14, 23, 113], and that ED predicts these behaviours in both ASC and BPD [114, 115].

In terms of gender differences, contrary to our hypothesis (H4), autistic women did not show a heightened ED compared to autistic men and to the BPD group. Indeed, the BPD group scored the highest compared to both autistic women and men. This result is inconsistent with findings supporting a heightened ED in autistic women compared to both autistic men [24, 25, 27] and women with BPD [27]. However, studies comparing ED in autistic women and people with BPD are scarce [27] and those comparing ED across genders in autistic adults have focused on individuals attending psychiatric facilities, unlike our study [25, 27, 53]. Thus, compared to these findings, our results are probably more representative of ED levels across genders in the adult autistic population irrespective of their co-occurring disorders. It is noteworthy, however, that autistic women had higher levels of anxiety and stress compared to autistic men, but lower scores than the BPD group. This is in line with findings supporting an increased vulnerability to anxiety in autistic women compared to autistic men, that may be attributed to psychosocial (e.g. increased use of camouflaging) and biological (e.g. heightened sensory sensitivity) factors [24, 116, 117].

As expected, the analyses between groups showed that the ASC group had the highest ASC traits (measured by the AQ-Short), while the BPD group showed the highest BPD traits (measured by the BSL-23). However, the ASC group scored higher than the NC group on the BSL-23 and the BPD group scored higher than the NC group on the AQ-Short, which might reflect the overlapping features between ASC and BPD [28, 118]. ADHD traits were equivalent between the ASC and BPD groups, but the ADHD co-occurrence was higher in ASC compared to BPD (34% in the ASC group and 24% in the BPD group). This could be due to an increased awareness among clinicians of the high co-occurrence between ADHD and ASC and their shared neurodevelopmental nature, which might increase the likelihood of screening for ADHD in autistic people [119, 120]. Moreover, alexithymia scores were found to be similar in our clinical groups, but also across genders in the ASC group. Hence, although alexithymia has been strongly linked to ASC traits [121], it can be rather seen as a transdiagnostic feature also found in BPD [122]. Interestingly, alexithymia has been found to be closely linked to ED regardless of diagnosis in clinical populations [123, 124]. However, despite being closely related, ED and alexithymia seem to be independent constructs. Indeed, if alexithymia fully accounted for ED, alexithymia scores would have been heightened in the BPD group compared to the ASC group, since ED was found to be increased in the former group. Yet such was not the case, as no difference was found between the two groups. Therefore, it is likely that other factors are particularly heightened in BPD and contribute to the increased ED scores found in this group.

Regarding factors specifically related to Linehan's biosocial model, the BPD group showed higher scores on the scales assessing emotional vulnerability and invalidating experiences compared to the ASC group. This is in line with the fact that ED is central in BPD, and the transaction between these components is recognized as predictive of ED in this disorder [22, 34]. Nevertheless, autistic women showed similar emotional vulnerability scores compared to the BPD group and tended to present with higher levels of emotional vulnerability relative to autistic men. The rates of sexual abuse in autistic women were also found to be equivalent to the BPD group in our study, and both were higher than those reported by autistic men and the NC group. These results add to findings pointing to an increased prevalence of sexual abuse among autistic women [125–127]. In fact, Cazalis et al. [125] reported a 2 to threefold increase of victimization in autistic women compared to non-autistic women, as well as high rates of revictimization. This, in turn, is associated with a higher risk to develop mental health issues, including anxiety disorders and PTSD [125, 128]. Additionally, autistic women showed higher scores on both the camouflaging and the sensory processing scales compared to both autistic men and the BPD group. However, no significant difference was found in sensory processing between autistic men and the BPD group. Given that sensory sensitivity has been linked to daily psychophysiological arousal and increased anxiety, this might partially explain why autistic women tended to present with higher emotional vulnerability and anxiety scores relative to autistic men in our study [23, 129].

Interestingly, while autistic women presented with increased emotional vulnerability and sensory sensitivity (biological components), and higher rates of sexual abuse and camouflaging (psychosocial components) than autistic men, it is worth noting that ED scores were equivalent between the two genders. This can be explained by the fact that autistic women may have increased emotion regulation abilities relative to autistic men, which might compensate for their heightened exposure to ED risk factors and to anxiety. Consistent with this hypothesis, autistic women have been reported to have increased social skills [130] and greater use of autistic camouflaging [68, 69] than autistic men, which could underlie a greater ability to engage in goal-directed behaviour that may be useful for emotion regulation although the selected goals, e.g. camouflaging, might be detrimental to their well-being on the long run [68, 69]. Previous data have reported an increased use of emotion regulation strategies in NC women compared to men, while ED is equally associated with psychopathology in both genders [131].

Considering the predictors of ED, we found that BPD traits, emotional vulnerability and alexithymia predicted ED in both clinical groups. Consistently, the SHAP value plots showed that the BPD traits were the main ED predictor across groups (ASC/BPD/NC), followed by emotional vulnerability and alexithymia. These results suggest that ED is central to BPD as conceptualized by Linehan’s theory [34]. Additionally, they indicate that BPD traits, emotional vulnerability and alexithymia might be central to ED, irrespective of the diagnosis. Interestingly, BPD traits were a stronger predictor of ED in the ASC group compared to the BPD group with a wider range of SHAP value. This can be explained by the fact that the BSL-23 (measuring BPD traits) might lack discriminative and predictive power in the BPD group, as BPD traits are intrinsic to BPD. Conversely, in ASC, it is likely that BPD traits may refer to the overlapping difficulties between ASC and BPD (e.g. social communication peculiarities, disturbed sense of self) [28, 118]. Moreover, emotional vulnerability was identified as one of the three strongest ED predictors in both groups, suggesting a strong biological basis underlying ED regardless of diagnosis. Alexithymia ranked second in the SHAP value plots for ASC, while emotional vulnerability ranked second for BPD. Thus, although alexithymia has been pinpointed by several studies as a transdiagnostic process involved in ED (e.g. [27, 132]), this suggests that alexithymia might be particularly central to ED in ASC. The latter result is in line with previous findings, suggesting that alexithymia is heightened in autistic women compared to women with BPD [27]. Although alexithymia does not belong among ASC core features [133], it has been closely associated with ASC in the literature [121]. Interestingly, recent research indicates that alexithymia might primarily arise from interoception awareness deficits, i.e. low ability to perceive the internal state of one’s body (e.g. fatigue, hunger, pain, temperature and heart rate) in both clinical and nonclinical populations [134, 135]. In ASC, impaired interoception has been reported in both autistic youth and adults [136, 137], and it has been associated with alexithymia [136]. In BPD, fewer studies have investigated interoception abilities, but similar impairments have been reported [138]. It is therefore possible that interoception deficits and alexithymia are more prominent in ASC. ASC-related factors, on the other hand, did not predict ED in the ASC group compared to the BPD group, contrary to our hypotheses (H5 and H6). However, sensory sensitivity showed a trend towards significance in the linear regression model in the ASC group and ranked fourth in the SHAP value plot. This suggests that sensory processing particularities may be the ASC-related factor that contributes the most to ED in ASC, which is congruent with previous findings in autistic youth [20, 139]. It is noteworthy that autistic camouflaging ranked as the fifth strongest ED predictor in the ASC group. In the BPD group, ADHD symptoms ranked as the fourth predictor in the SHAP value plot, which is consistent with the fact that impulsivity is a core feature of BPD [140].

Furthermore, contrary to our hypotheses (H5 and H6**)**, the childhood invalidation measures, including early trauma (CTQ-SF) and bullying (ABE), did not emerge as significant ED predictors in the clinical groups, which is inconsistent with Linehan’s model [34]. Another study also failed to identify parental invalidation as BPD predictors and suggested that it was irrelevant for the model [37], while Keng & Soh [41] suggested that maternal invalidation contributed to BPD. IT is noteworthy that Linehan [34] considered that invalidation included several forms of emotional invalidation (i.e. minimization, punishment, ignoring the emotional experience) with a focus on parental invalidation that was not tackled by the measures we used (i.e. CTQ-SF and the ABE). Moreover, in Linehan’s theory, it is rather the transaction between the two components that is key to ED, and this was not assessed in our study [34].

## Limitations

Our study has a number of limitations. First, the sample size (*N* = 724) was limited, especially since the participants were divided into three groups. Using a larger sample would have also allowed to optimize our predictive models for ED, especially those based on ML as large datasets are supported to improve the accuracy of such models [141, 142]. Second, as the study was conducted online, the ASC and BPD diagnoses were self-reported, which did not allow us to check their accuracy. However, it was stated in the online form that a formal diagnosis provided by a psychiatrist was required. Third, age was not homogeneous between groups, with the ASC group having the highest mean age (*M* = 32.62) (vs. *M* = 28.64 for the BPD group and *M* = 25.92 for the NC group). Compared to the NC group, the difference can be explained by the fact that 76% of this group were students (vs. 49% in the ASC group), since the study was advertised at several universities in France. Compared to the BPD group, the difference could be due to two factors: (i) the delayed diagnosis of autistic adults without intellectual disability (e.g. mean age at diagnosis of 34.1 in the study by Lehnhardt et al. [143]) with long delays between the first assessments and the diagnosis (e.g. an average of 8 years in the study by Gesi et al. [144]), and (ii) the possible attenuation of BPD symptoms over the lifespan reported in the literature [145, 146]. Fourth, we assessed self-harming and did not distinguish skin cutting from other forms of self-harm. Given that skin cutting in particular is has been associated with severe ED [20] and with suicidal behaviours [147], it would be relevant to distinguish between different types of self-harming behaviours in future studies. In addition, we note that rates of self-harm over the year prior to the study (35%) and lifetime suicidal behaviour (12%) were particularly high in the NC group. In fact, although research acknowledges the presence of these behaviours in the general population, the rates reported are lower than those found in our study (e.g. past-year self-harm of 6% and lifetime suicidal behaviour of 3% among a sample of college students in the study by Macrynikola et al. [148]). This may be explained by the relatively young age of the NC group. Indeed, consistent with the rates reported in our study, high prevalence rates of NSSI among adolescents and young adults have been reported in recent studies (e.g. 12-month prevalence rates between 15.5 and 31.3% in middle-income countries according to Aggarwal et al. [149]). Moreover, there has been a significant increase in the prevalence of NSSI in recent years, consistent with the rising rates of mental health problems among young people [150, 151]. The COVID-19 pandemic in particular has been linked to the increased rates of NSSI among the youngest populations [152]. Additionally, we note that the research theme may have attracted participants who felt concerned by the mental health issues exposed even though they did not have a psychiatric and/or a neurodevelopmental diagnosis. Fifth, all the study measures were self-reported. Indeed, self-report scales have numerous limitations, including relying on the participant’s ability to accurately self-assess their functioning [153]. This might be particularly challenging in studies on ED since it might be accompanied by high levels of alexithymia, which was the case in our study. Sixth, our autistic group was recruited in the general population and does not necessarily present with ED, whereas our BPD group presents with ED given that ED is a core feature in BPD. This limits our conclusions regarding the differential diagnosis in the specific case of autistic adults with severe ED associated with self-harm and/or suicidal behaviours comparable to those seen in BPD. Future studies should focus on the specific case of autistic adults with ED in comparison with BPD to identify clues that might inform the process of differential diagnosis, or even the identification of the co-occurrence, akin to the study by Weiner et al. [27]. It should be noted, however, that our study identified a number of potential areas for differential diagnosis between ASC and BPD within the ED framework, i.e. the potential involvement of sensory sensitivity and the camouflaging of autistic traits in autistic people with ED. Furthermore, our results suggest considering the potentially central implication of alexithymia in ASC compared to BPD. Seventh, participants who reported a diagnosis of ASC + BPD were not considered. Given that this subgroup is likely to present with specific characteristics, future studies should focus on the nature of the ED in adults with ASC + BPD. Eighth, although we have opted for a short version for each scale, we had many questionnaires, which probably explains the high dropout rate in our study (51%). Reducing the number of measures could lighten the burden on participants, reduce the dropout rate and potentially improve the quality of the data collected [154]. Ninth, in our study we did not explore the transactional and the potential moderating/mediating relationships between the model’s components and factors. Indeed, this study aimed to provide initial results of ED comparing ED correlates between BPD, ASC and a nonclinical group. These results may pave the way to further investigate how the different ED correlates identified for ASC interact. Tenth, our cross-sectional findings prevent any conclusions regarding the direction and causality of relationships between ED and its correlates; longitudinal research is warranted to address the latter. Finally, we measured the psychosocial factors of the model through the CTQ-SF (childhood trauma) and the ABE (school bullying), whereas there are tools that may be more appropriate to measure invalidation as conceptualized by Linehan [34], such as the Socialization of Emotion Scale [92].

## Conclusions

To conclude, ED scores are higher in ASC compared to nonclinical controls, but milder than in BPD. While gender did not predict ED scores in our sample, autistic women had increased risk factors to ED relative to autistic men (i.e. emotional vulnerability, sexual abuse, sensory particularities, autistic camouflaging and anxiety). This suggests that it is crucial to consider gender-related factors potentially involved in ED in ASC in future studies. Importantly, the same three dimensions, i.e. BPD traits, alexithymia and emotional vulnerability, seem to be involved in ED across the clinical groups, suggesting that they might be key to ED irrespective of diagnosis. However, sensory processing and autistic camouflaging may be more specific to ED in ASC, while ADHD symptoms may play a specific role in ED in BPD. Given that the same three main contributors to ED were found in ASC and BPD, our results outline that DBT, built upon Linehan’s biosocial model, is likely to be as relevant to treat ED in autistic adults as it is in BPD.

### Supplementary Information

**Additional file 1**.

## Data Availability

The datasets used and/or analysed during the current study are available from the corresponding author on reasonable request.

## Abbreviations

ABE: Assessment of bullying experiences
ADHD: Attention deficit hyperactivity disorder
AQ-Short: Autism spectrum quotient short version
ASC: Autism spectrum condition
ASRS v1.1 Screener: ADHD self-report scale v1.1 screener
BPD: Borderline personality disorder
BSL-23: Short form of the borderline symptom list
CAT-Q: Camouflaging autistic traits questionnaire
CBT: Cognitive behavioural therapy
cPTSD: Complex post-traumatic stress disorder
CTQ-SF: Childhood trauma questionnaire-short form
DASS-21: Depression, anxiety and stress scales
DBT: Dialectical behaviour therapy
DERS-16: Difficulties in emotion regulation scale-16
EV-Child: Emotional vulnerability-child scale
GAFS-8: Eight‑item general alexithymia factor score
M: Mean
ML: Machine learning
NC: Nonclinical controls
OLS: Ordinary least square
SD: Standard deviation
SHAP: Shapley additive explanation
SPSQ-SS: Sensory processing sensitivity questionnaire-sensory sensitivity subscale
ToM: Theory of mind

## Footnotes

1 Throughout the manuscript, we use the term “autistic people” (identity-first), rather than "people with autism" or "people with autism spectrum disorder", as this was the terminology explicitly favoured by the majority of the autistic participants of a large-scale survey [155]. We also use “autism spectrum condition” instead of the DSM-5 term of “autism spectrum disorder (ASD)” to be respectful to those on the spectrum who feel that the term “disorder” is stigmatizing, whereas the term “condition” acknowledges the difficulties, the differences and strengths in autistic people [156].
