# Supplementary material for: The biosocial correlates and predictors of emotion dysregulation in autistic adults compared to borderline personality disorder and nonclinical controls
Source: Mol Autism. 2023 Dec 18;14:47. doi: 10.1186/s13229-023-00580-3 (PMC10726572; doi:10.1186/s13229-023-00580-3)

The biosocial correlates and predictors of emotion dysregulation in autistic adults compared to borderline personality disorder and nonclinical controls

Correlation matrixes for each group

BPD group

	DERS-16	AQ-Short	BSL-23	EV-Child	CAT-Q	CTQ-SF	GAFS-8	SPSQ	ASRS v1.1 Screener	ABE	Gender
**DERS-16**	1.000	0.151	0.601	0.518	0.290	0.213	0.473	0.179	0.417	0.369	0.000
**AQ-Short**	0.151	1.000	0.152	0.187	0.440	0.180	0.065	0.311	0.214	0.347	0.227
**BSL-23**	0.601	0.152	1.000	0.407	0.229	0.255	0.407	0.009	0.354	0.394	0.345
**EV-Child**	0.518	0.187	0.407	1.000	0.397	0.302	0.130	0.255	0.348	0.327	0.198
**CAT-Q**	0.290	0.440	0.229	0.397	1.000	0.238	0.203	0.308	0.339	0.348	0.079
**CTQ-SF**	0.213	0.180	0.255	0.302	0.238	1.000	0.032	0.199	-0.000	0.453	0.101
**GAFS-8**	0.473	0.065	0.407	0.130	0.203	0.032	1.000	0.078	0.206	0.148	0.123
**SPSQ**	0.179	0.311	0.009	0.255	0.308	0.199	0.078	1.000	0.228	0.166	0.000
**ASRS v1.1 Screener**	0.417	0.214	0.354	0.348	0.339	-0.000	0.206	0.228	1.000	0.326	0.256
**ABE**	0.369	0.347	0.394	0.327	0.348	0.453	0.148	0.166	0.326	1.000	0.138
**Gender**	0.000	0.227	0.345	0.198	0.079	0.101	0.123	0.000	0.256	0.138	1.000

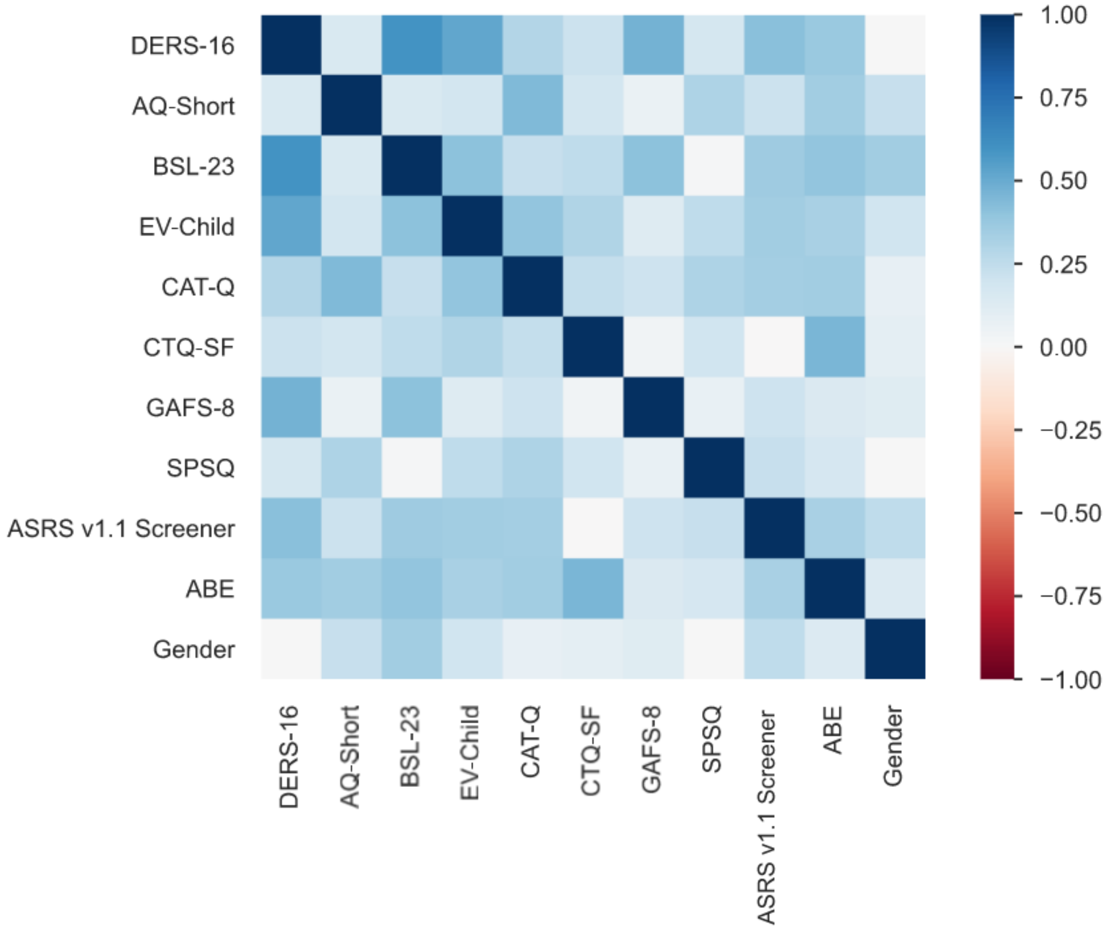

ASD group

	DERS-16	AQ-Short	BSL-23	EV-Child	CAT-Q	CTQ-SF	GAFS-8	SPSQ	ASRS v1.1 Screener	ABE	Gender
**DERS-16**	1.000	0.237	0.632	0.421	0.324	0.218	0.437	0.302	0.295	0.219	0.000
**AQ-Short**	0.237	1.000	0.034	0.123	0.225	0.241	0.335	0.075	0.038	0.111	0.000
**BSL-23**	0.632	0.034	1.000	0.319	0.236	0.253	0.278	0.204	0.326	0.256	0.197
**EV-Child**	0.421	0.123	0.319	1.000	0.351	0.334	0.113	0.363	0.118	0.450	0.000
**CAT-Q**	0.324	0.225	0.236	0.351	1.000	0.312	0.170	0.225	0.192	0.243	0.000
**CTQ-SF**	0.218	0.241	0.253	0.334	0.312	1.000	0.214	0.228	0.256	0.436	0.000
**GAFS-8**	0.437	0.335	0.278	0.113	0.170	0.214	1.000	0.017	0.197	0.103	0.068
**SPSQ**	0.302	0.075	0.204	0.363	0.225	0.228	0.017	1.000	0.208	0.214	0.000
**ASRS v1.1 Screener**	0.295	0.038	0.326	0.118	0.192	0.256	0.197	0.208	1.000	0.275	0.000
**ABE**	0.219	0.111	0.256	0.450	0.243	0.436	0.103	0.214	0.275	1.000	0.104
**Gender**	0.000	0.000	0.197	0.000	0.000	0.000	0.068	0.000	0.000	0.104	1.000

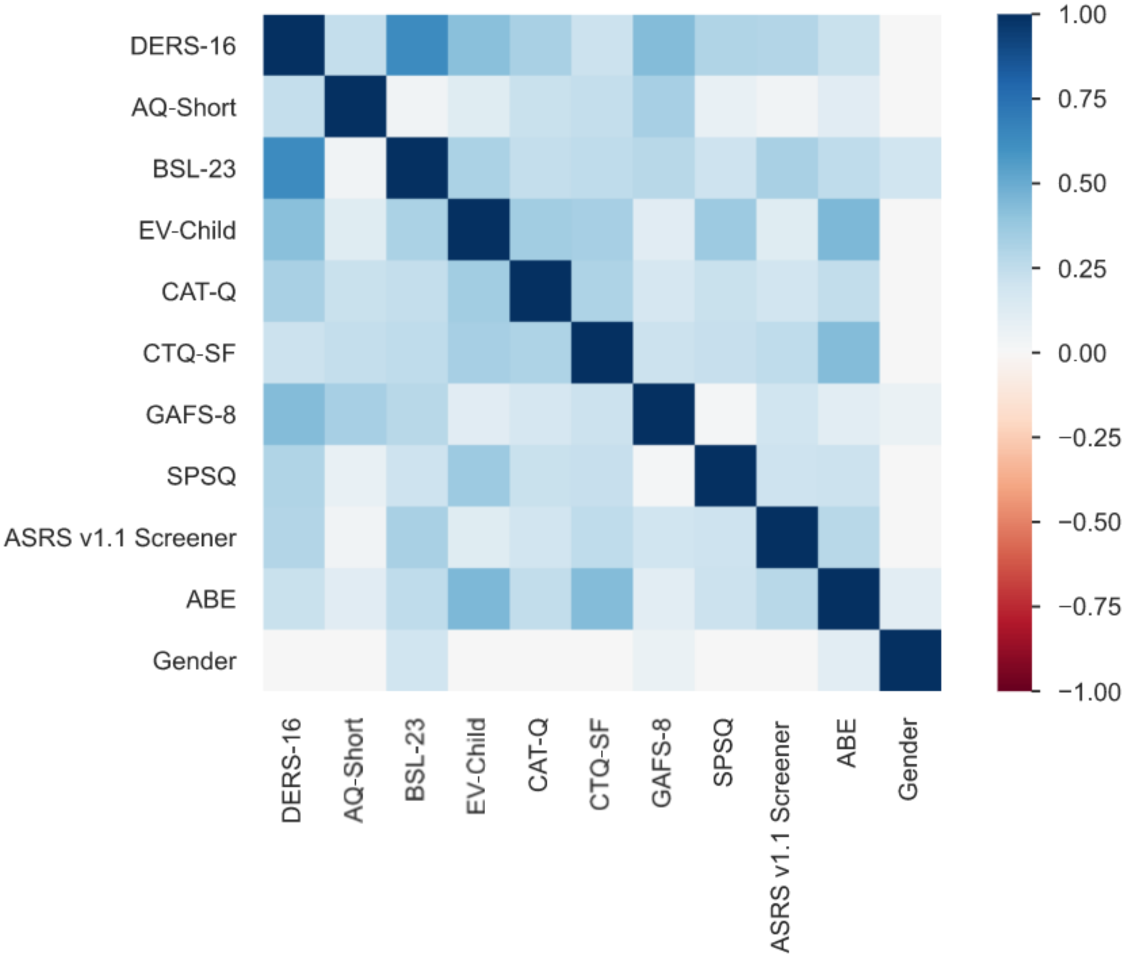

<u>ASD women group</u>

	DERS-16	AQ-Short	BSL-23	EV-Child	CAT-Q	CTQ-SF	GAFS-8	SPSQ	ASRS v1.1 Screener	ABE
**DERS-16**	1.000	0.246	0.618	0.349	0.322	0.158	0.429	0.273	0.281	0.146
**AQ-Short**	0.246	1.000	0.013	0.003	0.209	0.138	0.298	0.166	0.177	-0.000
**BSL-23**	0.618	0.013	1.000	0.290	0.300	0.228	0.298	0.097	0.266	0.215
**EV-Child**	0.349	0.003	0.290	1.000	0.293	0.366	0.124	0.380	0.004	0.434
**CAT-Q**	0.322	0.209	0.300	0.293	1.000	0.302	0.207	0.290	0.108	0.189
**CTQ-SF**	0.158	0.138	0.228	0.366	0.302	1.000	0.217	0.136	0.193	0.360
**GAFS-8**	0.429	0.298	0.298	0.124	0.207	0.217	1.000	0.034	0.203	0.052
**SPSQ**	0.273	0.166	0.097	0.380	0.290	0.136	0.034	1.000	0.131	0.226
**ASRS v1.1 Screener**	0.281	0.177	0.266	0.004	0.108	0.193	0.203	0.131	1.000	0.199
**ABE**	0.146	-0.000	0.215	0.434	0.189	0.360	0.052	0.226	0.199	1.000

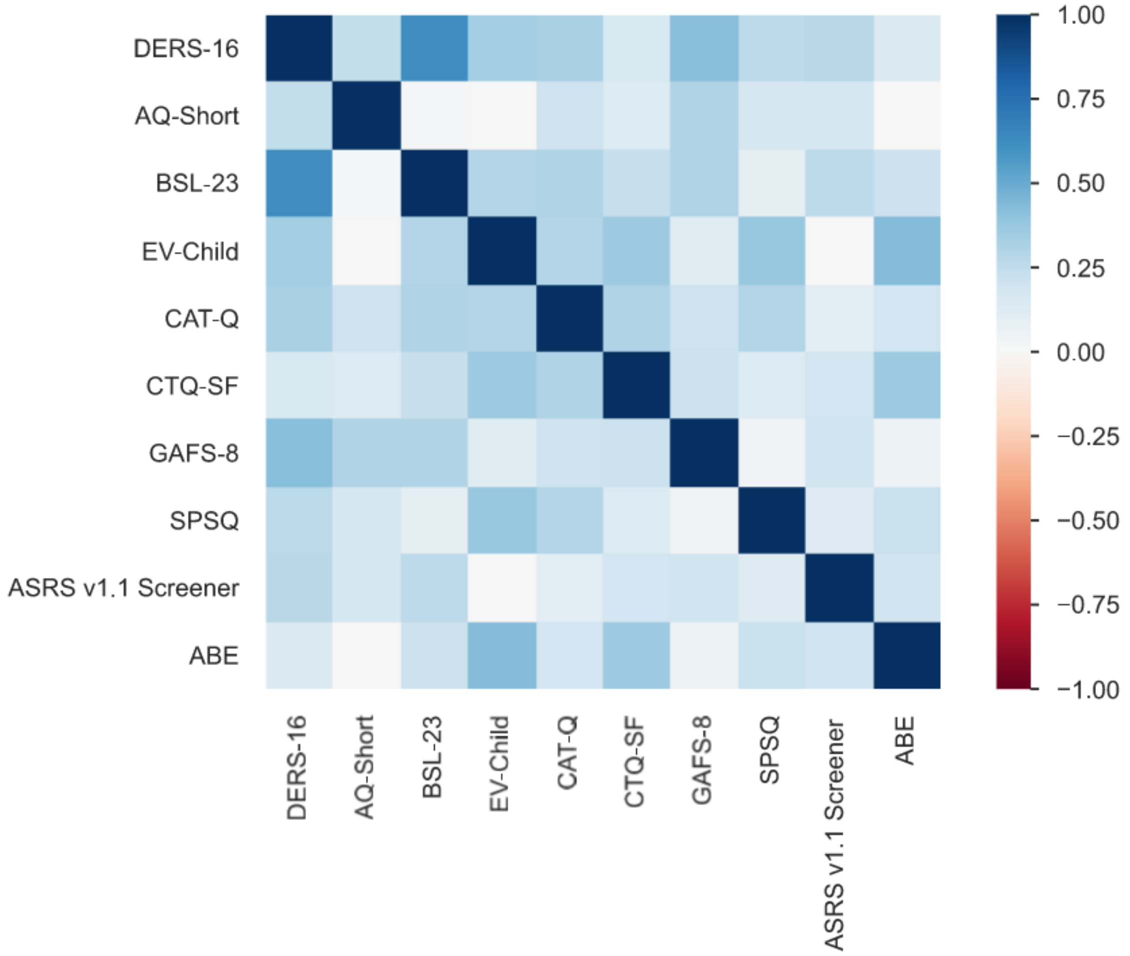

ASD men group

	DERS-16	AQ-Short	BSL-23	EV-Child	CAT-Q	CTQ-SF	GAFS-8	SPSQ	ASRS v1.1 Screener	ABE
**DERS-16**	1.000	0.295	0.630		0.499	0.152	0.238	0.228	0.249	0.282
**AQ-Short**	0.295	1.000	0.128	0.386	0.268	0.394	0.327	0.129	-0.150	0.273
**BSL-23**	0.630	0.128	1.000	0.319	-0.004	0.223	0.434	0.104	0.269	0.250
**EV-Child**	0.499	0.386	0.319	1.000	0.305	0.165	0.176	0.296	0.229	0.432
**CAT-Q**	0.152	0.268	-0.004	0.305	1.000	0.163	0.027	0.028	0.259	0.256
**CTQ-SF**	0.238	0.394	0.223	0.165	0.163	1.000	0.243	0.440	0.177	0.380
**GAFS-8**	0.626	0.327	0.434	0.176	0.027	0.243	1.000	0.107	0.221	0.220
**SPSQ**	0.228	0.129	0.104	0.296	0.028	0.440	0.107	1.000	0.228	0.280
**ASRS v1.1 Screener**	0.249	-0.150	0.269	0.229	0.259	0.177	0.221	0.228	1.000	0.255
**ABE**	0.282	0.273	0.250	0.432	0.256	0.380	0.220	0.280	0.255	1.000

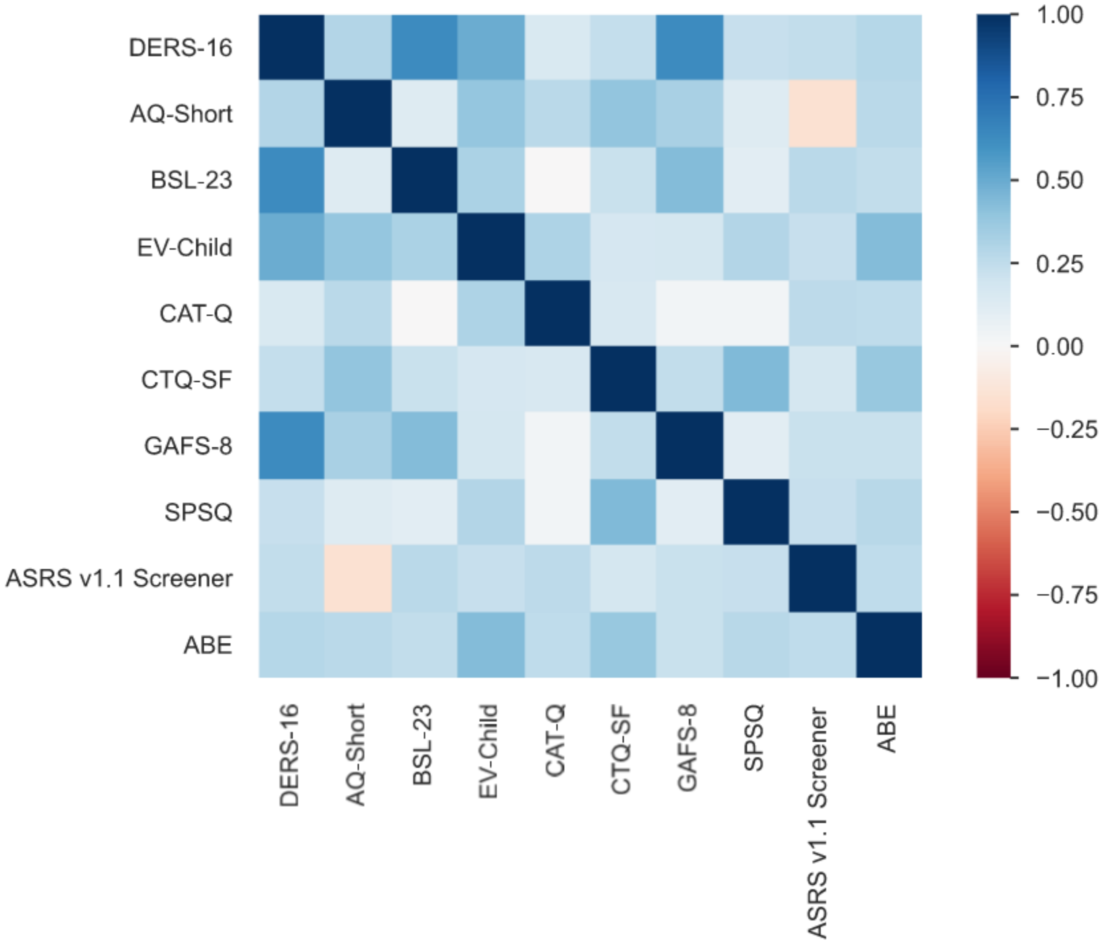

NC group

	DERS-16	AQ-Short	BSL-23	EV-Child	CAT-Q	CTQ-SF	GAFS-8	SPSQ	ASRS v1.1 Screener	ABE	Gender
**DERS-16**	1.000	0.429	0.742	0.532	0.393	0.323	0.576	0.343	0.500	0.335	0.079
**AQ-Short**	0.429	1.000	0.421	0.311	0.529	0.317	0.479	0.219	0.375	0.295	0.209
**BSL-23**	0.742	0.421	1.000	0.451	0.431	0.409	0.502	0.275	0.487	0.373	0.222
**EV-Child**	0.532	0.311	0.451	1.000	0.363	0.317	0.246	0.401	0.339	0.375	0.000
**CAT-Q**	0.393	0.529	0.431	0.363	1.000	0.291	0.419	0.303	0.396	0.349	0.272
**CTQ-SF**	0.323	0.317	0.409	0.317	0.291	1.000	0.225	0.167	0.292	0.437	0.087
**GAFS-8**	0.576	0.479	0.502	0.246	0.419	0.225	1.000	0.159	0.396	0.250	0.192
**SPSQ**	0.343	0.219	0.275	0.401	0.303	0.167	0.159	1.000	0.179	0.143	0.000
**ASRS v1.1 Screener**	0.500	0.375	0.487	0.339	0.396	0.292	0.396	0.179	11.000	0.256	0.143
**ABE**	0.335	0.295	0.373	0.375	0.349	0.437	0.250	0.143	0.256	1.000	0.133
**Gender**	0.079	0.209	0.222	0.000	0.272	0.087	0.192	0.000	0.143	0.133	1.000

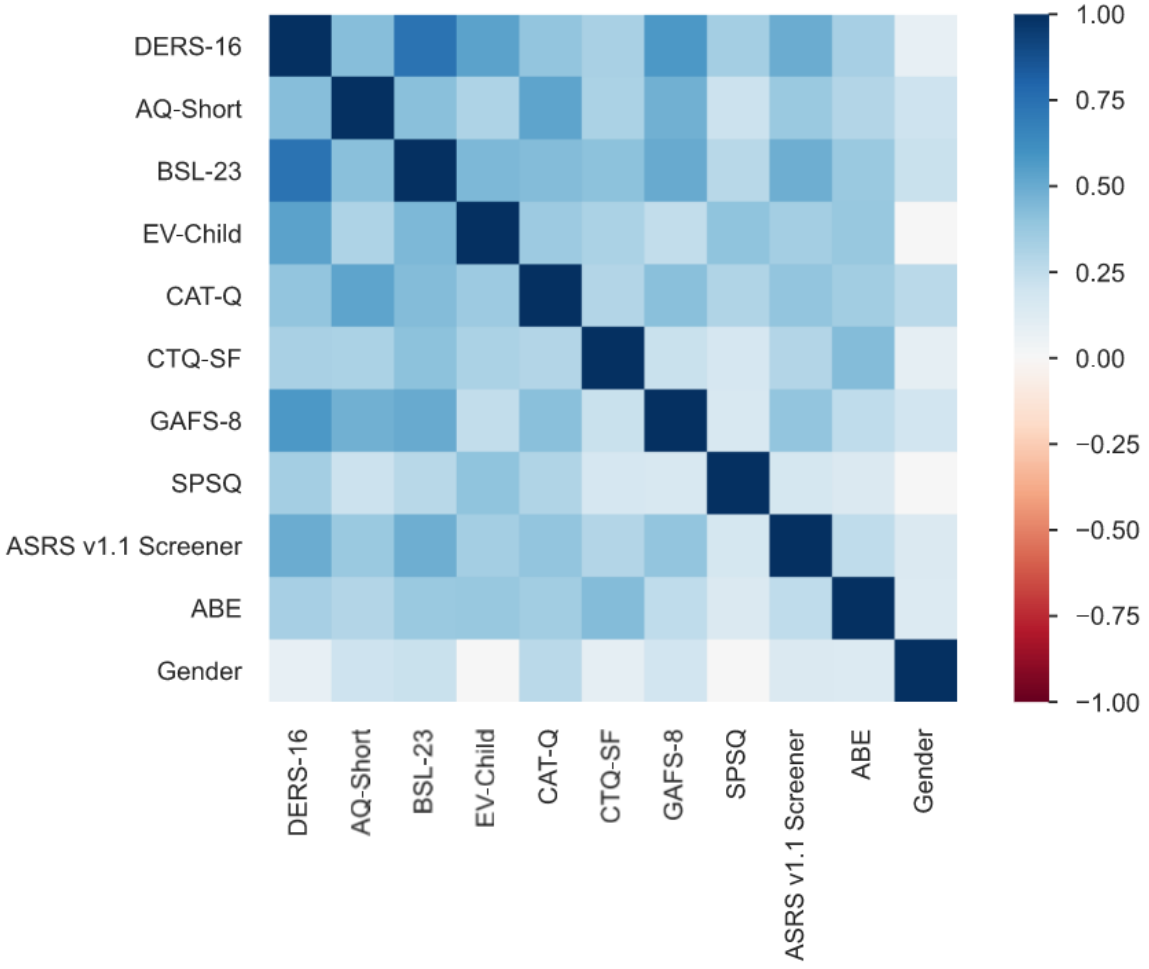

Supplement: Supplementary file 1 — Additional file 1. [file 13229_2023_580_MOESM1_ESM.pdf]
